# Enhancing Efficiency in Multi-Stage Pharmaceutical Manufacturing: A Process-Based Network DEA Approach

**DOI:** 10.12688/f1000research.166387.1

**Published:** 2025-07-18

**Authors:** Syarifa Hanoum, Mahmood Shubbak

**Affiliations:** 1 Department of Business Management, Institut Teknologi Sepuluh Nopember, Surabaya, 60111, Indonesia; 2 Department of Management, College of Economics and Political Science, Sultan Qaboos University, Muscat, Muscat Governorate, 123, Oman

**Keywords:** Network Data Envelopment Analysis, NDEA, Performance Measurement, Efficiency, Process Improvement, Decision-Making, Pharmaceutical Industry.

## Abstract

**Background:**

Manufacturing inefficiencies result in substantial financial losses for global industries. The present study introduces a robust Performance Measurement System (PMS) incorporating Network Data Envelopment Analysis (NDEA) to address efficiency challenges in multi-stage manufacturing systems.

**Methods:**

The study employs a case study approach within the pharmaceutical industry to reveal the pragmatic application of NDEA, which serves as the primary analytical instrument for evaluating performance across diverse production stages. Focusing on the production processes of intravenous (IV) sets, the research aims to highlight how NDEA disaggregates interconnected processes and quantify efficiency measures to pinpoint sources of inefficiencies in particular production stages and actionable insights for operational improvement.

**Results:**

First, the NDEA-based PMS provides insights to address specific process inefficiencies on the shop floor, providing strategic insights for process improvement. Second, despite its power in pinpointing the source of inefficiency, modelling a process-based PMS faces a challenge as increasing the number of stages in the model presents a trade-off between the accuracy and discrimination power of the NDEA model.

**Conclusions:**

This investigation contributes to the literature by proposing a data-driven, process-based PMS framework combining methodological rigor and practical applicability. The proposed framework provides managers with actionable guidance to optimize performance and enhance operational efficiency in complex manufacturing settings. The novel framework establishes a pivotal resource for strategic decision-making and fosters process innovation.

## Introduction

In today’s rapidly evolving business landscape, the fierce competition for innovative products and services necessitates a focus on uniqueness to achieve success in both domestic and international markets ( Aziz et al., 2022; Handri et al., 2021, Shubbak, 2018). As companies strive to differentiate themselves, they face the daunting reality that manufacturing inefficiencies drain billions of dollars from global industries annually. This financial burden stresses the pressing necessity for robust performance measurement tools, which are essential for boosting competitiveness and nurturing the seeds of innovation necessary for future success ( Mio et al., 2022). Performance measurement is a vital process for organizations seeking to evaluate efficiency and effectiveness, aligning operational activities with strategic goals ( Dahinine et al., 2024; Melnyk et al., 2014; Neely et al., 2002; Pujotomo et al., 2022). In the manufacturing sector, where process efficiency is critical, Performance Measurement Systems (PMS) are pivotal in tracking, evaluating, and enhancing performance, to ensure the production process is in a cost-effective manner ( Hanoum, 2021; Hanoum & Islam, 2021; Rodríguez et al., 2024). Manufacturing firms should go above and beyond to achieve their strategic goals and increase their performance ( Azizi et al., 2025; Handri et al., 2021).

Despite their recognized significance, current PMS frameworks frequently fail to deliver practical guidance for operationalizing performance indicators at the process level. Established models, such as the Balanced Scorecard ( Kaplan & Norton, 1996), the Baldrige Excellence Framework (BEF) ( Arif, 2007), and the European Foundation for Quality Management (EFQM) model ( Doeleman et al., 2014), highlight strategic alignment but often lack comprehensive methodologies for implementing performance measures in complex, multi-stage manufacturing contexts ( Neely et al., 2002; Van Looy & Shafagatova, 2016). The current PMS frameworks also lack guidance on how process performance measures are chosen and operationalised in practice.

This gap underscores the need for frameworks that align with strategic objectives and provide in-depth operational processes tailored to specific environments. Additionally, it underlines the need for advanced analytical tools that quantify efficiency and deliver detailed insights into specific process-related inefficiencies. Network Data Envelopment Analysis (NDEA) presents a robust framework by representing manufacturing operations as interlinked processes, thus capturing inputs and outputs across multiple stages of production. Unlike conventional Data Envelopment Analysis (DEA), which often treats production systems as monolithic entities or ‘black boxes’, NDEA disaggregates these systems. This enables a more granular examination of inefficiencies, allowing for the identification of targeted areas for enhancement ( Färe & Primont, 1984; Tone & Tsutsui, 2009).

While existing research has demonstrated the utility of NDEA for benchmarking and efficiency evaluation, there remains a lack of studies applying NDEA within single enterprises to develop a process-based PMS ( Castelli et al., 2010; Kao, 2014; Rachmad et al., 2024). Additionally, the impact of increasing stages on the discrimination power of NDEA models remains underexplored. Addressing these gaps, this study investigates the following research questions:

▪ The practical application of NDEA in real-world settings faces numerous challenges ( J. S. Liu et al., 2013; Paradi & Sherman, 2014). To address this, the study asks: How can a NDEA-based PMS be practically implemented to improve multi-stage manufacturing processes? **(RQ1).**
▪ Several DEA studies have analysed the internal structure of Decision-Making Units (DMUs) and found that NDEA has a stronger discrimination power compared to classical DEA ( Castelli et al., 2010). This study aims to verify and expand on this theory by posing the question: How does the number of stages in a NDEA model affect its discrimination power? **(RQ2).**

To answer these questions, this article presents a case study focused on the production line of a pharmaceutical company’s intravenous (IV) sets, exemplifying the intricacies of multi-stage manufacturing, featuring a combination of manual and automated processes. The study introduces a framework that integrates Network Data Envelopment Analysis (NDEA) into a Performance Management System (PMS), providing a systematic methodology for assessing, quantifying, and enhancing operational performance.

The structure of the article is organized as follows: the next section provides a literature review on NDEA, tracing its evolution from DEA to NDEA, discussing fundamental concepts, and detailing the selection of NDEA models. A section on methodology follows this, presenting the stages of the research. The subsequent section presents a case study that illustrates the application of NDEA within an IV sets production line, in a pharmaceutical company. Following this, we explore the proposed practical NDEA-based performance management system (PMS) derived from the case study. The concluding sections summarize the key findings, draw conclusions, and make recommendations for future research.

## Literature review: Network data envelopment analysis

### From DEA to NDEA

Data Envelopment Analysis (DEA) is a widely used methodology for evaluating efficiency across decision-making units (DMUs) based on inputs and outputs. However, its “black-box” approach often fails to capture the complexities of multi-stage systems where intermediate outputs are significant ( Färe & Primont, 1984; Seiford & Zhu, 1999). To address this limitation, Network Data Envelopment Analysis (NDEA) extends DEA by modelling the internal structure of DMUs, providing a more granular assessment of efficiency ( Tone & Tsutsui, 2009). NDEA offers unique advantages over classical DEA, including the ability to disaggregate multi-stage processes into sub-processes, enabling a detailed evaluation of inefficiencies ( Färe & Primont, 1984), and improve strategic decision-making by identifying performance bottlenecks across stages ( Kao, 2014). Table 1 summarizes key theoretical advancements of NDEA compared to DEA.

**Table 1.** The key theoretical advancements of NDEA compared to DEA.

Aspect	DEA	NDEA
System Structure	Treats DMUs as “black boxes” without internal process analysis.	Models interconnected sub-processes within DMUs.
Intermediate Outputs	Does not account for intermediate products or services.	Explicitly incorporates intermediate outputs between stages.
Application Scope	Primarily used for single-stage or static systems.	Tailored for multi-stage, dynamic manufacturing systems.

### The Basic Concept of NDEA

**Figure 1.**
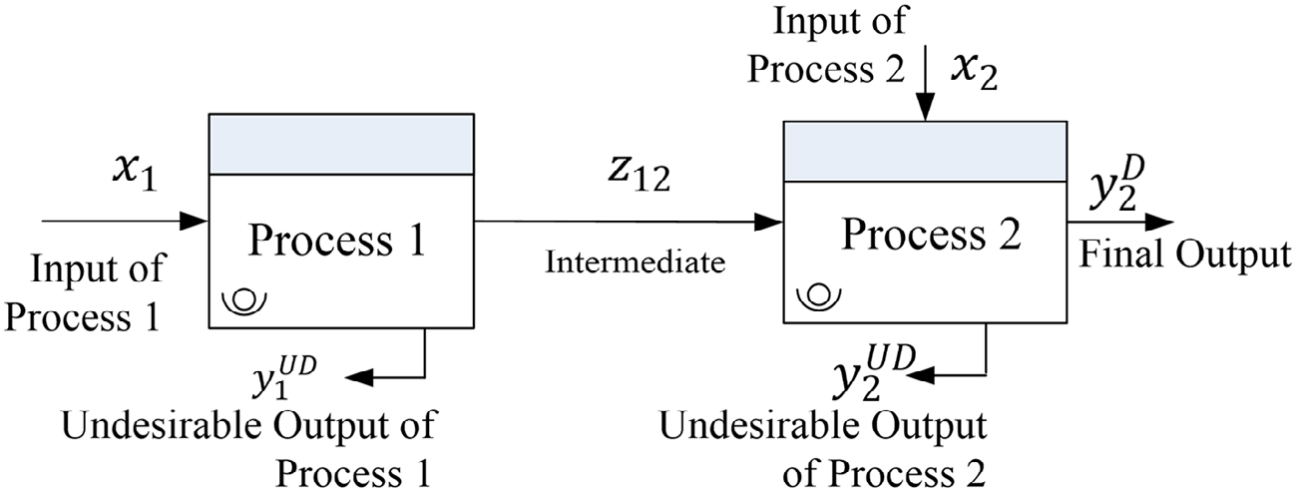
Interaction among input, output, and intermediate factors in two-stage manufacturing operations. The figure illustrates the flow of desirable and undesirable outputs, including intermediate products (z12) between two processes, and the role of inputs (x1, x2) and final output (y2).

( Färe & Primont, 1984) initiated the exploration of the ‘black-box’ system of classical DEA and was followed by other scholars ( Seiford & Zhu, 1999; Wang et al., 1997). Figure 1 illustrates the interactions among inputs (x _i_), outputs (y _r_), and intermediate factors (z _kh_) in two-stage manufacturing operations. S _(k,h)_ represents the number of intermediate measures passing from the k ^th^ process to the h ^th^ process. Process 1 has input (x _1_) and the output of process 1, which is called intermediate output (z _12_), becomes the input in process 2. In addition to z _12_, process 2 has a supplementary input (x _2_) to yield output (y _2_). Given that process 2 is the final process, y _2_ is the final output of the manufacturing operations.

While the intermediate and final products are characterized as desirable outputs, a manufacturing process often generates undesirable outputs including rejected products and waste. Figure 1 presents the undesirable outputs produced by both processes (y _1_^UD^ and y _2_^UD^). Given that desirable outputs are expected to be maximized, undesirable outputs must be minimized. Some authors have incorporated undesirable outputs in the DEA model ( Chung et al., 1997; Scheel, 2001).

### Selections of the NDEA models

(a) Distance, orientation, and scale assumptions

Researchers may choose the NDEA models between the radial and non-radial approaches. The radial approach works under the proportionality assumption in the changes of inputs or outputs. Because in manufacturing operations, production factors such as labour, materials, and capital may not be changed proportionally, we choose the network slacks-based measure (NSBM) that operates the efficiency measurement based on input excess and output shortfall ( Tone & Tsutsui, 2009). The choice of orientation between input and output depends on whether the decision-makers’ focus is on controlling inputs or outputs to improve efficiency. We chose a non-oriented approach to avoid any subjective preferences and merely rely on the mathematical model to assess those production factors or outputs that should be improved. In NDEA, two-scale assumptions are commonly employed: constant returns to scale (CRS) and variable returns to scale (VRS).

In DEA literature, the combination of non-orientation, non-radial, and VRS modelling is often used to enhance the relevance of frontier efficiency studies ( Avkiran, 2011). VRS is often highlighted for accommodating the differing economies of scale among Decision-Making Units (DMUs), where scale is not constant in nature ( Zuniga-Gonzalez et al., 2025). However, this study evaluates a manufacturing line across various production periods where economies of scale are not a concern. Therefore, we selected the combined NSBM, non-oriented, CRS model as the most suitable NDEA approach for multi-stage manufacturing operations within a single plant.

(b) The presence of undesirable outputs

NDEA models maximise the outputs of a system to achieve the system’s optimum efficiency. Manufacturing environments, however, may generate undesirable outputs such as production wastes, rejected products, and pollution. Therefore, NDEA, which incorporates undesirable outputs in its mathematical model, is considered. We applied this undesirability phenomenon to the NSBM approach ( W. B. Liu et al., 2010) as shown in the mathematical model 1 below. The extended model is characterised as NSBM-non-oriented-CRS with undesirable outputs.

$$\begin{array}{c} \mathrm{Min}\;\rho_{o}^{*}=\frac{\sum_{k=1}^{K}W^{k}\left[1-\frac{1}{m_{k}}\left(\sum_{i=1}^{m_{k}}\frac{s_{i}^{k-}}{x_{\mathrm{io}}^{k}}\right)\right]}{\sum_{k=1}^{K}W^{k}\left[1+\frac{1}{R_{k}^{D}+R_{k}^{\mathrm{UD}}}\left(\left(\sum_{r_{k}^{D}=1}^{R_{k}^{D}}\frac{S_{r}^{k{+}^{D}}}{y_{\mathrm{ro}}^{k^{D}}}\right)+\left(\sum_{r_{k}^{\mathrm{UD}}=1}^{R_{k}^{\mathrm{UD}}}\frac{S_{r}^{k{+}^{\mathrm{UD}}}}{y_{\mathrm{ro}}^{k^{\mathrm{UD}}}}\right)\right)\right]}\\ \text{Subject to}\\ \sum_{j=1}^{n}x_{\mathrm{ij}}^{k}\lambda_{j}^{k}-S_{i}^{k-}\leq x_{\mathrm{io}}^{k},\;i=1,\ldots ,m_{k},\;k=1,\ldots ,K\;\\ \sum_{j=1}^{n}y_{r_{k}^{D}j}^{k^{D}}\lambda_{j}^{k}-S_{r}^{k{+}^{D}}=y_{r_{k}^{D}o}^{k^{D}},\;r_{k}^{D}=1,\ldots ,R_{k}^{D},\;k=1,\ldots ,K\\ \sum_{j=1}^{n}y_{r_{k}^{\mathrm{UD}}j}^{k^{\mathrm{UD}}}\lambda_{j}^{k}-S_{r}^{k{+}^{\mathrm{UD}}}=y_{r_{k}^{\mathrm{UD}}o}^{k^{\mathrm{UD}}},\;r_{k}^{\mathrm{UD}}=1,\ldots ,R_{k}^{\mathrm{UD}},\;k=1,\ldots ,K\\ \sum_{j=1}^{n}\sum_{s_{\left(k,h\right)}=1}^{S_{\left(k,h\right)}}z_{s_{\left(k,h\right)}j}^{\left(k,h\right)}\lambda_{j}^{k}=\sum_{j=1}^{n}\sum_{s_{\left(k,h\right)}=1}^{S_{\left(k,h\right)}}z_{s_{\left(k,h\right)}j}^{\left(k,h\right)}\lambda_{j}^{h},\;\forall \left(k,h\right),\;\lambda_{j}^{k},S_{r}^{k+}\geq 0 \end{array}$$ (1)

The study’s sample size would be n DMUs (j = 1, …, n), which consist of K sub-processes (k = 1, …, K). The objective $\rho_{o}^{*}$ characterises the non-oriented efficiency score of DMUo (the subscript “o” represents the DMU under analysis), while $w^{k}$ in this function is denoted as a subjective weight of the kth sub-process. Because the subjectivity is avoided in this study’s decision-making process, the weights are set as 1.00 for all sub-processes. If $\rho_{o}^{*}=1$ and all input and output slacks are equal to zero, then the DMUo is efficient. The method decomposes the overall efficiency into divisional/process efficiency ( $\rho_{k}$ ), given in model (2), where $s_{i}^{k-*}$ and $s_{r}^{k+*}\;$ are the optimal input-slack and output-slack for model (1). All nomenclatures of models (1) and (2) are described in Table 2.

$$\rho_{k}=\frac{1-\frac{1}{m_{k}}\left(\sum_{i=1}^{m_{k}}\frac{s_{i}^{k-*}}{x_{\mathrm{io}}^{k}}\right)}{1+\frac{1}{r_{k}}\left(\sum_{r=1}^{r_{k}}\frac{s_{r}^{k+*}}{y_{\mathrm{ro}}^{k}}\right)},\;k=1,\ldots ,K\;$$ (2)

**Table 2.** The nomenclature of model 1.

Subscript “ *o*” is related to the DMU which is under observation		
$\rho_{o}^{*}$	=	The overall non-oriented efficiency score of DMU _o_.
$w^{k}$	=	The weight of the *k*th process/division determined by decision-makers.
$x_{\mathrm{ij}}^{k}$	=	The *i* ^th^ input, *i* = 1, …, m _k_, which corresponds to the *k* ^th^ ( *k* = 1, …, *K*) process of the *j* ^th^ DMU ( *j* = 1, …, *n*).
$y_{\mathrm{rj}}^{k}$	=	The *r* ^th^ output, *r*= 1, …, r _k,_ which corresponds to the *k* ^th^ ( *k* = 1, …, *K*) process of the *j* ^th^ DMU ( *j* = 1, …, *n*).
$S_{r}^{k+}$	=	Amount of slack related to the *r* ^th^ output of the *k* ^th^ ( *k* = 1, …, *K*) process.
$z_{s_{\left(k,h\right)}j}^{\left(k,h\right)}$	=	An intermediate factor from the *k* ^th^ process to the *h* ^th^ process ( *k* ≠ *h* and *k*, *h* = 1, …, K).
$\lambda_{j}^{k}$	=	The intensity vector corresponding to the *k* ^th^ process of the *j* ^th^ DMU.
*n*	=	Number of DMUs.
*K*	=	Number of processes/divisions.
$m_{k}$	=	Number of inputs corresponding to the *k* ^th^ process.
$r_{k}^{D}$	=	The subscript corresponding to desirable outputs of the divisions/processes.
$R_{k}^{D}$	=	Number of desirable outputs corresponding to the *k* ^th^ process.
$r_{k}^{\mathrm{UD}}$	=	The subscript corresponding to undesirable outputs of the divisions/processes.
$R_{k}^{\mathrm{UD}}$	=	Number of undesirable outputs corresponding to the *k* ^th^ process.
$s_{\left(k,h\right)}$	=	The subscript of intermediate measure from the *k* ^th^ process to *h* ^th^ process.
$S_{\left(k,h\right)}$	=	Number of intermediate measures passing from the *k* ^th^ process to *h* ^th^ process.
$\rho_{k}$	=	The divisional/process efficiency of the *k* ^th^ process.

## Methods

The research begins with a comprehensive literature review to identify the most appropriate model. The options include radial and non-radial methods, between constant returns to scale (CRS) or variable returns to scale (VRS). The model’s orientation can be input-oriented, output-oriented, or non-oriented. This study selected the combined non-radial Non-SBM (NSBM) non-oriented CRS model as the most suitable NDEA method for analyzing multi-stage manufacturing processes within a single plant.

The next phase of the study involves case study research, where we apply the selected NDEA model to a pharmaceutical company. This application highlights the unique challenges of developing NDEA-based PMS for assessing performance. The results of the case study led to recommendations for managers, identifying sources of inefficiency and targets for process improvement. Additionally, we deliver a practical framework, derived from insights gained during the case study research, designed to assist managers in implementing NDEA within the multi-stage manufacturing process.

### Applying NDEA to measure performance of a pharmaceutical production process: A case study

This case study illustrates the transformation of manufacturing operations by applying the NDEA model. It explores the practical implementation of NDEA to establish a multi-stage Performance Measurement System (PMS) within a pharmaceutical production environment. The study aims to achieve two main objectives: developing a practical, process-based performance measurement framework for single-enterprise, multi-stage systems, and examining the trade-off between NDEA model complexity and discrimination power.

The pharmaceutical company occupies approximately 60,000 square meters. It promotes four product groups: intravenous sets (IV Sets), IV Solutions, Therapeutic Drugs, and Clinical Nutrition. The company dominates the market with a 70% share in basic solutions and medicines, catering to both domestic and international markets. Its operations adhere to high regulatory standards and stringent quality requirements, making efficiency and waste minimization crucial for maintaining competitiveness.

This study focuses on the IV Set production line, a medical device used for transferring nutrients intravenously or conducting blood transfer. The strategic choice of this line is based on several reasons:

▪ **Strategic Importance:** IV sets represent one of the company’s highest-revenue product lines, accounting for nearly 30% of total sales. Ensuring optimal performance in this line has a direct impact on overall profitability.
▪ **Operational Complexity:** The production of IV sets involves combination of machining and manual processes, which present operational challenges for executives when managing performance fluctuations.
▪ **Process Variability:** The IV-set line frequently experiences performance fluctuations due to machine downtime, material inconsistencies, and manual assembly errors. These challenges underscore the need for a robust performance measurement framework to identify inefficiencies and guide process improvements.
▪ **Scalability and Relevance:** Insights from the IV-set line can be generalized to other product lines within the company, as many share similar multi-stage production structures.

### Process mapping

**Figure 2.**
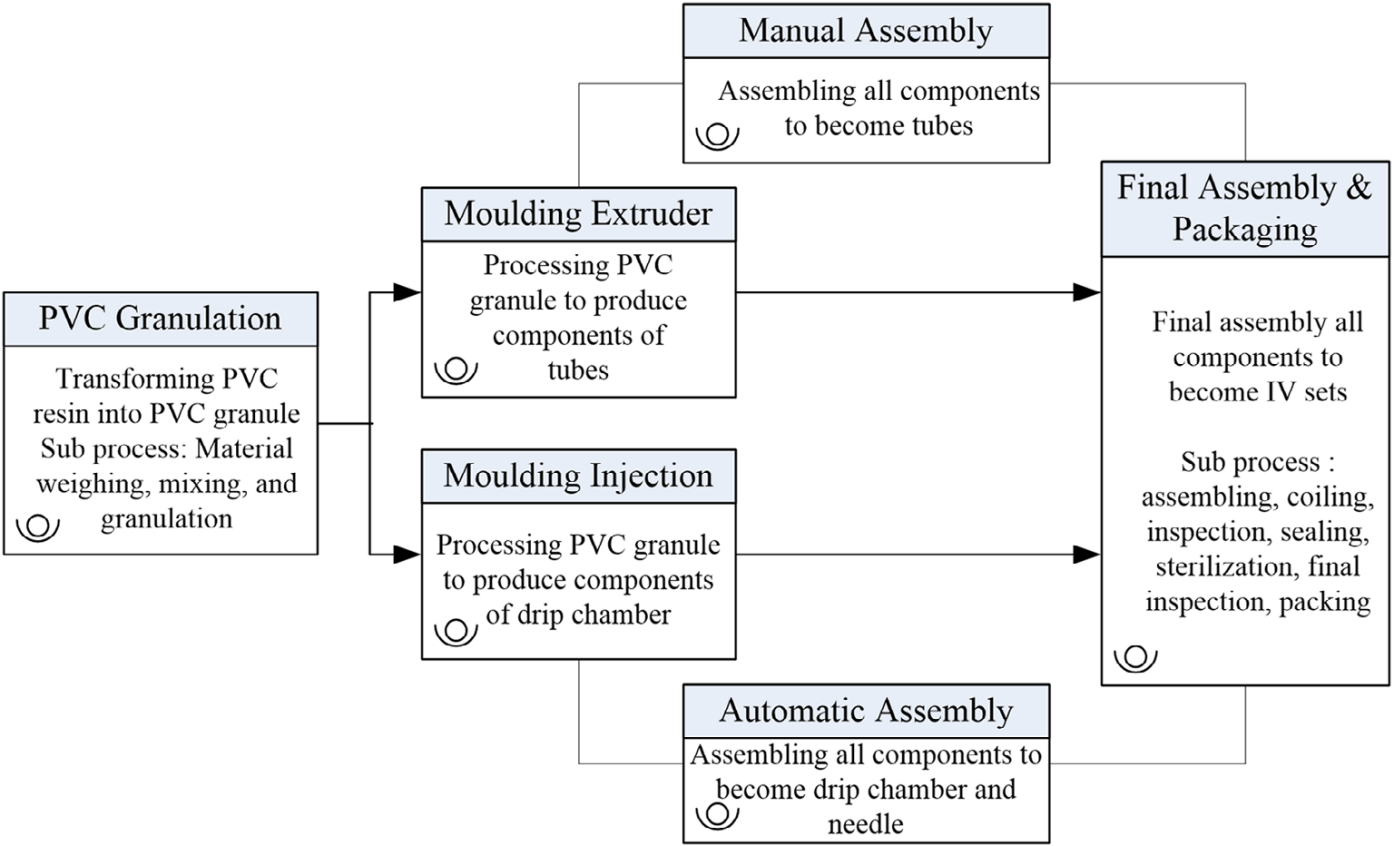
Process map of the intravenous (IV) set production process. This diagram outlines six interconnected workstations: PVC Granulation, Moulding Extruder, Moulding Injection, Manual Assembly, Automatic Assembly, and Final Assembly & Packaging.

IV-sets are produced across six interconnected workstations, as depicted in Figure 2. These stages include:

**PVC Granulation (WS1):** Conversion of raw Polyvinyl Chloride (PVC) resins into medical-grade granules.

▪ **Moulding Extruder (WS2):** Fabrication of sub-assembled tubes using the granulated PVC.
▪ **Moulding Injection (WS3):** Production of components such as drip chambers, spikes, and needle covers.
▪ **Manual Assembly (WS4):** Manual combination of sub-assembled tubes and additional components.
▪ **Automatic Assembly (WS5):** Automated assembly of finished IV-set components, including drip chambers.
▪ **Final Assembly and Packaging (WS6):** Integration of all components, final quality checks, and packaging.

Each stage is interdependent, with outputs from one stage serving as inputs for the next. This multi-stage structure makes the IV-set line an ideal case for applying NDEA, which accounts for intermediate outputs and interconnected processes.

### NDEA variables and model specification

In NDEA studies, model specification is crucial to list the performance assessment criteria. However, studies that have rationalized the essential variables for performance assessment are scarce ( Cook et al., 2014; Kishore et al., 2024). Once the process map is available, identifying inputs and outputs for each process becomes more straightforward when adopting the NDEA for manufacturing operations.

The initial process involves consuming various PVC resins as raw materials to produce non-toxic medical-grade PVC granules. These granules serve as inputs for the subsequent moulding processes. In the second stage, moulding includes additional materials to create a range of components. The moulding extruder generates an assortment of sub-assembled tubes, whereas the moulding injection yields drip chamber components (e.g., the central part of drip chambers, spikes, needle covers, joints, and seals) for the following assembly process. Both PVC granulation and moulding processes require operators and machines.

The assembly processes combine all the parts produced by the prior workstations. The assembly station comprises three sub-stations: automatic assembly, manual assembly, and final assembly. The automatic assembly combines components from moulding injection into the finished unit of drip chambers. It is characterized as a one-man-one-machine workstation, where machine-hour or man-hour is used interchangeably. As for the manual assembly workstation, sub-assembled tubes from the moulding extruder and additional components from suppliers are manually assembled, requiring only man-hour as the input. The final assembly line involves a combination of machine and manual work, signifying man-hours and machine-hours.

Outputs from the earlier stages, which function as inputs for the subsequent stages, are called intermediate factors. The last stage generates the final outputs. The ultimate product of this production line is the IV-Set in various sizes intended for the transfer of nutrition, medication, and blood. In addition, the IV-Set production line yields waste or rejected outputs known as undesirable outputs. Another undesirable output refers to machine downtime that occurs in PVC granulation, moulding extruder, and moulding injection workstations. Meanwhile, the assembling activities that utilize machines and equipment with lower breakdown risks render machine downtime - an insignificant factor in all assembly processes.

A favourable DEA/NDEA model is typically characterized by its discrimination power. The discrimination power of a DEA model can be compromised when a massive number of inputs and outputs are used, mainly because a particular number of DMUs is considered efficient in certain scenarios ( Cook et al., 2014). As a rule of thumb, the number of DMUs should be at least twice the total number of inputs and outputs. Adhering to this guideline minimizes the correlation between variables and DEA/NDEA outputs, thereby enhancing the discriminating power of the model.

**Figure 3.**
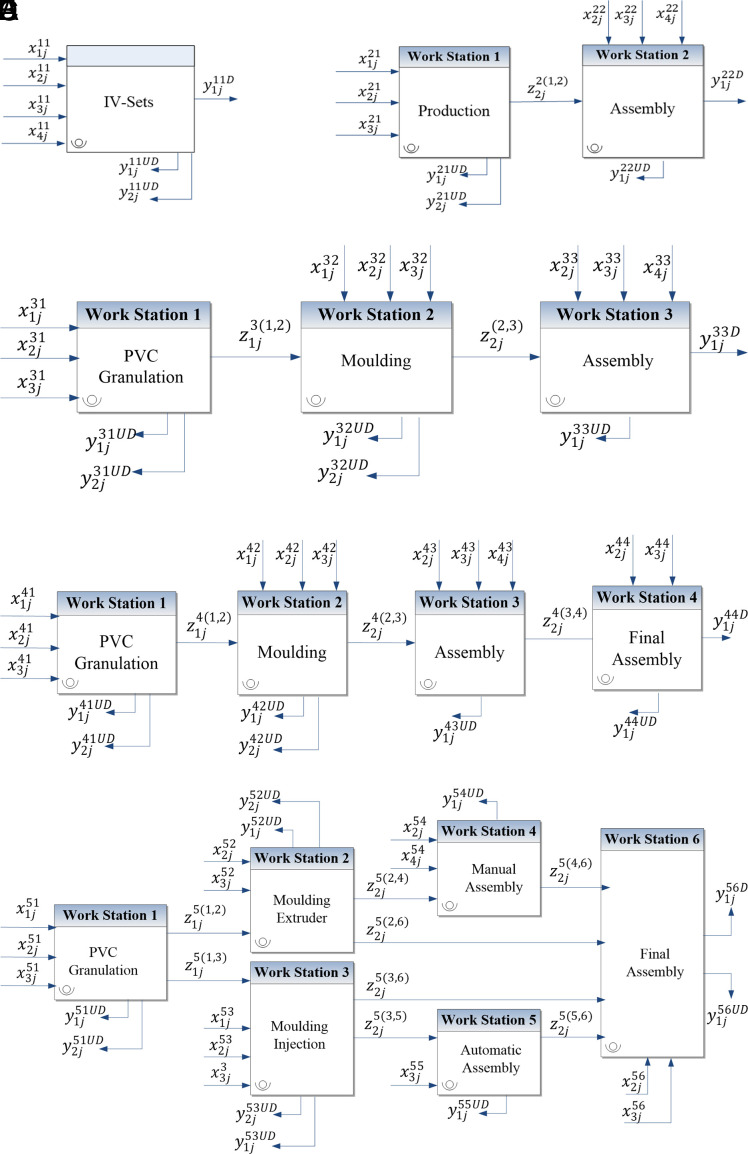
Interaction of inputs, intermediate factors, and outputs in five NDEA models. The five subfigures represent different decomposition levels of the production system: (A) classical DEA, (B) two-stage NDEA, (C) three-stage NDEA, (D) four-stage NDEA, and (E) six-stage NDEA.

According to ( Castelli et al., 2010), discrimination power is higher in the NDEA model, compared to the classical model. In certain cases, it is possible for most or even all DMUs to be deemed inefficient. However, the idea that adding more stages to the NDEA model improves discrimination power is inconclusive. The existing literature does not provide clear guidance on how to divide a system into multi-stage and interconnected sub-systems, nor does it specify the optimal number of stages needed for particular manufacturing operations. To bridge these gaps, this paper outlines five scenarios aimed at identifying the most appropriate NDEA model for the IV-Set production system (refer to Figure 3). The nomenclature related to Figure 3 can be found in Table 3.

**Table 3.** The NDEA data corresponding to Figure 3.

Variables	Definitions	Unit of measures
$x_{1j}^{\mathrm{lk}}$	Raw materials corresponding to the *k* ^th^ process of the *j* ^th^ DMU.	Kilograms (kgs)
$x_{2j}^{\mathrm{lk}}$	Man-hour corresponding to the *k* ^th^ process of the *j* ^th^ DMU.	Hours (hrs)
$x_{3j}^{\mathrm{lk}}$	Machine-hour corresponding to the *k* ^th^ process of the *j* ^th^ DMU.	hrs
$x_{4j}^{\mathrm{lk}}$	Additional components corresponding to the *k* ^th^ process of the *j* ^th^ DMU.	Pieces (pcs)
$y_{1j}^{\text{lkUD}}$	Rejected outputs (undesirable output) corresponding to the *k* ^th^ process of the *j* ^th^ DMU.	kgs
$y_{2j}^{\text{lkUD}}$	Machine downtime (undesirable output) corresponding to the *k* ^th^ process of the *j* ^th^ DMU.	hrs
$z_{1j}^{l\left(k,h\right)}$	Intermediate outputs from the *k* ^th^ process to the *h* ^th^ process ( *k* ≠ *h* and *k*, *h* = 1, …, K).	kgs
$z_{2j}^{l\left(k,h\right)}$	Intermediate outputs from the *k* ^th^ process to the *h* ^th^ process ( *k* ≠ *h* and *k*, *h* = 1, …, K).	pcs
$y_{1j}^{\mathrm{lkD}}$	Final output (desirable output) corresponding to the *k* ^th^ process of the *j* ^th^ DMU.	pcs
*L*	Numerator index corresponding to the NDEA model/scenario under evaluation ( *l* = 1, …, 5).	

Referring to Figure 3, Model (1) illustrates the classical DEA, where the production line is a ‘black-box’ system and the intermediate factors are dismissed. Model (2) shows the simplest form of NDEA (two-stage NDEA), where the manufacturing process of IV-Set is divided into production and assembly stages. In model (2), the intermediate factors flow between the production and the assembly stages.

Model (3) expands on Model (2) by dissecting the production stage into PVC granulation and moulding after considering the intermediate factors and undesirable outputs that flow between the three stages. Unlike Model (2), Model (3) treats the additional materials required for moulding as a separate variable. Model (4) segregates the production system into four stages based on the responsibilities of the four supervisors in the IV-Set department. Each supervisor oversees one of the following four units: PVC granulation, moulding, assembly, and final assembly.

Model (5) incorporates the six workstations outlined in the process map. It looks into the internal structure of all processes, which consist of the largest number of stages and NDEA variables. The technical correctness of each model was validated by conducting a comprehensive review by the research team and production supervisors. The focus of this validation is to ascertain that there was no significant process change throughout the one-year study period to maintain the high acceptability of the models.

### Data collection and model selection

The following sub-section details the data source, the model selections based on five models, and the decision support system facilitated by NDEA for performance evaluation and process improvement. The data from 12 months, including inputs, outputs, and intermediate variables, were extracted from the company. The one-year monthly production period served as the DMUs to meet the requirements of the annual review conducted by the corporate board.

The NDEA efficiency scores were computed for each production month, hence treating the IV-Set production system as a one-, two-, three-, four-, or six-stage production process. The objective is to identify a model that aligns with NDEA requirements and serves as a decision support system for process improvement. The models must exhibit an acceptable level of discrimination power and offer accurate insights for process enhancement. The basic descriptive statistics shown at the bottom of Table 4 demonstrate how effectively the models differentiated the efficiency levels among the DMUs.

**Table 4.** Efficiency scores for NDEA model scenarios.

NO	DMU	1-stage	2-stage	3-stage	4-stage	6-stage
1	01_23	1	1	1	1	1
2	02_23	0.376	0.526	0.518	0.571	1
3	03_23	0.173	0.228	0.290	0.289	0.292
4	04_23	0.136	1	1	1	1
5	05_23	0.180	0.324	0.313	0.325	0.343
6	06_23	0.383	0.411	0.443	0.459	0.418
7	07_23	1	1	1	1	1
8	08_23	1	1	1	1	1
9	09_23	0.590	0.693	0.642	0.613	1
10	10_23	1	1	1	1	1
11	11_23	1	1	1	1	1
12	12_23	0.323	0.343	0.469	1	1
Average efficiency score		0.597	0.710	0.723	0.771	0.838
Least efficiency score		0.136	0.228	0.290	0.289	0.292
Standard deviation		0.347	0.306	0.294	0.291	0.295
Number of efficient DMUs		5	6	6	7	9

Apart from assessing whether an increased number of stages in NDEA can enhance discrimination power, examining the five models determined the most suitable model for the IV-Set production line. The discrimination power of NDEA was assessed by looking into the following statistics: average efficiency score, least efficiency score, number of efficient DMUs, and standard deviation, with a smaller value indicating greater discrimination power.

The descriptive statistics revealed that Model (1) performed best in distinguishing the efficiency scores throughout the production period. However, selecting a single-stage DEA model is unsuitable for a process-based PMS that emphasizes internal processes. This approach provided insufficient information to uncover the specific stages and production network that demands improvements. Model (5) was the favoured model for process enhancement because it offered detailed insights by breaking down the production line into more processes than the other models. However, more stages in an NDEA model introduce additional variables that may affect discrimination power. Based on Table 3, Model (5) exhibited the lowest discrimination power, as indicated by the highest average and the least efficiency values, the smallest standard deviation, and the highest number of efficient DMUs.

Upon comparing Models (1), (2), and (3), the statistical results disclosed that Model (3) had lower discrimination power than Models (1) and (2) for two reasons. First, Model (3) recorded a higher average efficiency score and the lowest efficiency score when compared to Models (1) and (2). Second, Model (3) had a smaller standard deviation value than the other two. Nonetheless, the oversimplification inherent in the single- and two-stage Models (1) and (2) limited their efficacy in comprehending the production system.

After considering the trade-offs, Model (3) was selected as the preferred model for several reasons. Given its moderate number of stages, Model (3) strikes a balance between the discrimination power required by NDEA and the necessary details for process improvement purposes. From the stance of PMS, Model (3) aligns with the parsimony principle, which emphasizes data collection and processing without excessive cost and time implications.

### Efficiency scores and peer groups

The NDEA non-parametric method measures efficiency by assessing each criterion measure (weighted output/input) and constructing an envelopment frontier across all measures to ascertain that the observed data points lie on or below the frontier. The three-stage NDEA model ( Figure 3C) was deployed in this case study. Scores were computed based on the 12-month production period. The efficiency scores (see Table 5) revealed that 50% of the production period fell below the production frontier. Table 5 presents the inefficient production months and their respective peer groups. A peer group refers to the efficient months with the most similar circumstances to each inefficient month concerning the input and output sets. For example, the peer group of production period 02_23 includes 07_23 and 11_23.

**Table 5.** The efficiency score of the three-stage NDEA-based PMS.

Work-stations	Efficient DMUs						Inefficient DMUs					
	01_23	04_23	07_23	08_23	10_23	11_23	02_23	03_23	05_23	06_23	09_23	12_23
Overall	1	1	1	1	1	1	0.518	0.290	0.313	0.443	0.642	0.469
PVC	1	1	1	1	1	1	0.475	0.332	0.327	0.512	0.782	0.599
Moulding	1	1	1	1	1	1	0.629	0.340	0.332	0.628	0.735	0.443
Assembly	1	1	1	1	1	1	0.665	0.395	0.471	0.394	0.612	0.674

Peer groups (efficient months)		07_23 11_23	07_23	07_23	07_23 11_23	07_23 11_23	07_23
Descriptive statistics of the production system							
	Average efficiency score	Least efficiency score	Standard Deviation				
Overall	0.723	0.290	0.303				
PVC	0.752	0.327	0.284				
Moulding	0.759	0.332	0.277				
Assembly	0.768	0.394	0.258				

The classical DEA displayed the sources of inefficiency through input and output variables. Taking a step further, the NDEA method evaluated each stage along the network to determine the production process with the most significant impact on the overall efficiency of the manufacturing operations. More insights were captured from the NDEA results, particularly by examining the descriptive statistics (see bottom of Table 4). The PVC granulation stage recorded the lowest efficiency score, which solidified its status as the most inefficient stage and a prominent contributor to the overall inefficiency of the IV-Set manufacturing line. The standard deviation of its efficiency score was the largest, translating into considerable performance fluctuations over the studied production year. On the contrary, the assembly stage displayed the highest average efficiency score and minimal performance variability, further confirming its pivotal role in bolstering the overall production efficiency.

### Process improvement

Referring to the efficiency scores, the NDEA produced slacks for each variable in the model to signify the shortfall of outputs or the excess of inputs that rendered a DMU inefficient. Besides, the NDEA offered improvement targets for each production factor (i.e., material, man-hour, and machine-hour) and output (i.e., machine downtime, rejected outputs, and good products). Improvement can manifest as a decrease in inputs and undesirable outputs or an increase in desirable outputs.

**Figure 4.**
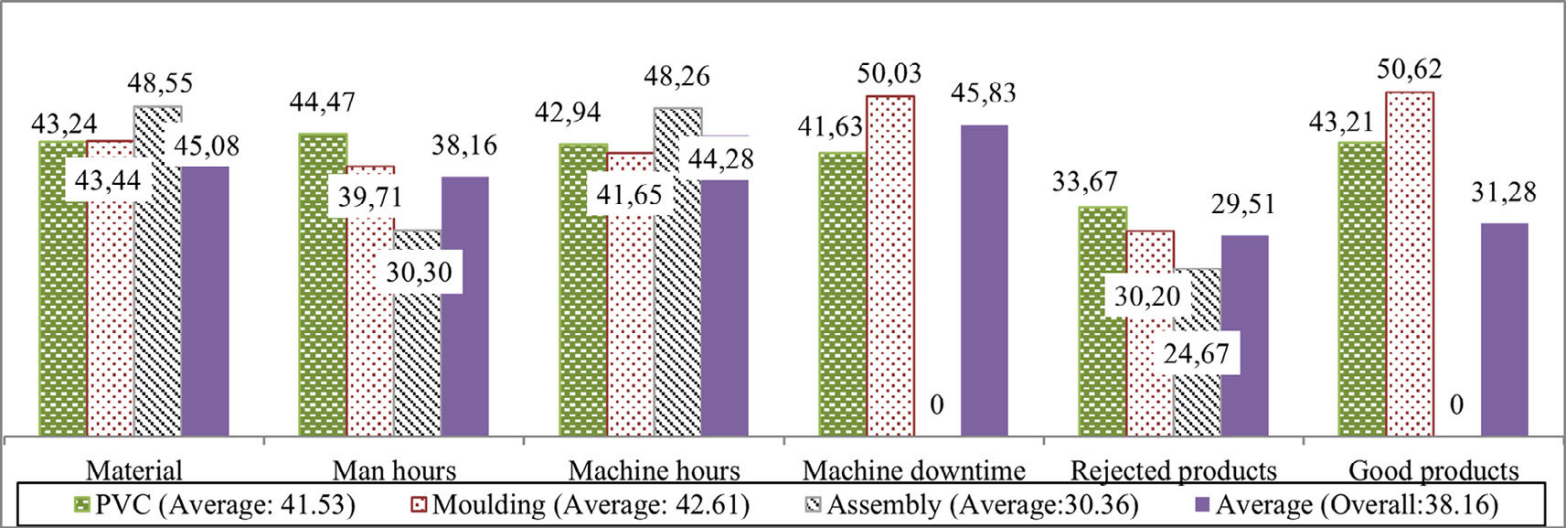
Percentage of potential improvement for the production process. This bar chart visualizes potential reductions in input materials, man-hours, machine-hours, and undesirable outputs, as well as the necessary increase in good outputs to achieve optimal efficiency.

For each category in Figure 4, the first, second, and third bars represent inputs consumed or outputs generated at the PVC granulation, moulding, and assembly workstations. The fourth bar depicts the average potential improvement required for each category.

Figure 4 illustrates the imperative need for the company to minimize input materials, man-hours, and machine-hours by 45.08%, 38.16%, and 44.28%, respectively. Moreover, a reduction of 45.83% for machine downtime and 29.51% for rejected outputs/waste appears to be crucial. To achieve 100% efficiency for the entire production system, a comprehensive approach involving cutbacks in all input factors and undesirable outputs, along with an increment of 31.28% in good outputs, is essential.

### The proposed framework of NDEA-based PMS

Managers find the NDEA-based PMS to be effective in analysing the performance fluctuations of production factors and outputs in each production process. This sheds light on the impact of such fluctuations on the overall performance of the production line. Both the production manager and supervisors concurred that the NDEA model comprehensively addressed the essential measures related to medical device manufacturing operations. The model facilitated identifying inefficient processes and pinpointed the production factors or outputs that required enhancement. Decisions associated with process enhancement typically fall in the purview of the manufacturing head department or production line managers and supervisors.

**Figure 5.**
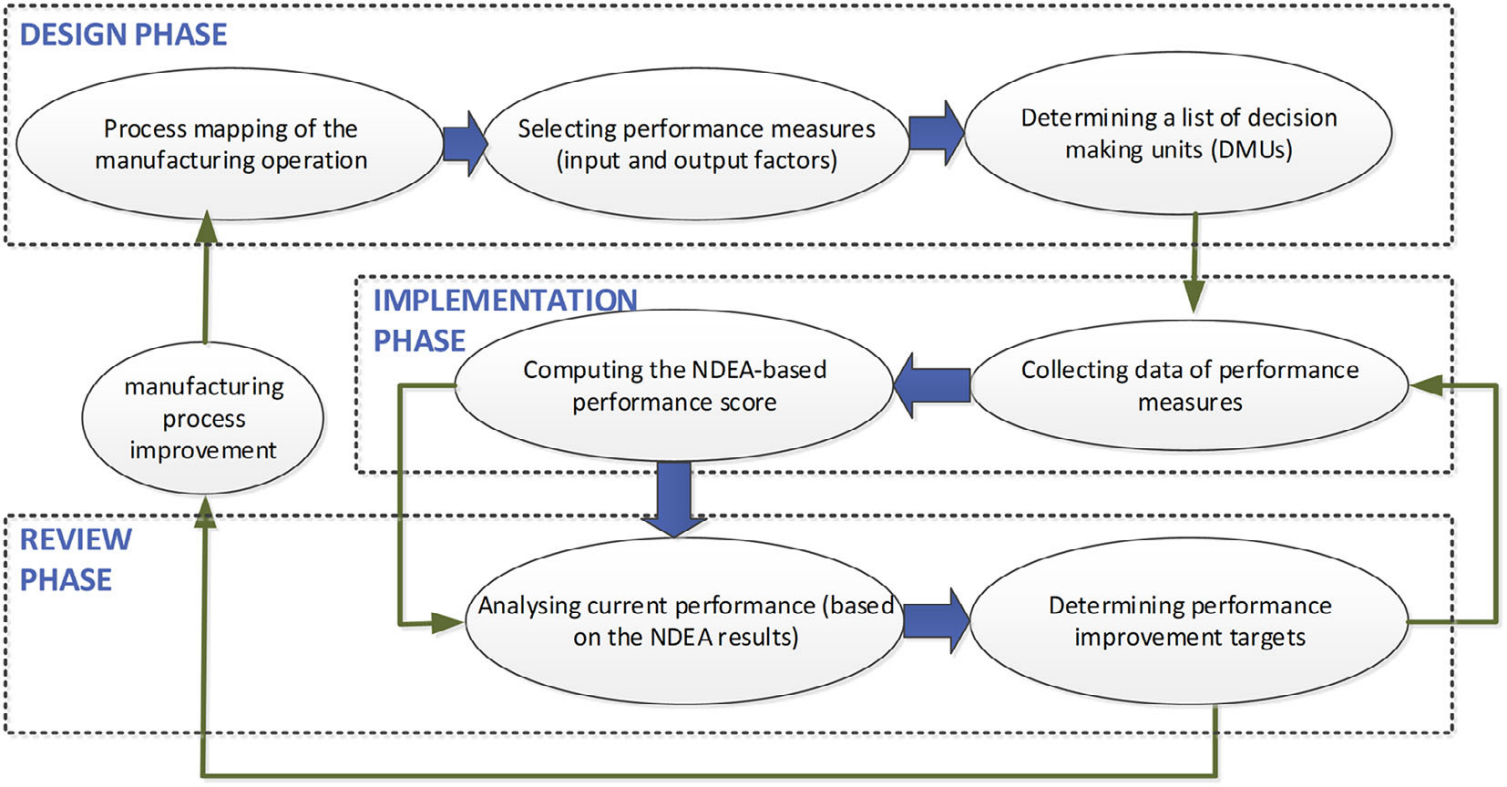
Proposed NDEA-based PMS framework. The diagram outlines three main phases: Design (process mapping and model selection), Implementation (data collection and scoring), and Review (adjustments and benchmarking).

A post-study meeting with the company’s executive board emphasized the need for a generic framework to extend DEA applications in manufacturing. A generalized framework is essential for managers applying similar techniques across production lines or manufacturing companies. Aligning with PMS principles, the proposed framework consists of three main phases: design, implementation, and review (see Figure 5).

In the design phase, the initiation involves process mapping, outlining the systematic flow of the manufacturing process from raw materials to finished products ( Lindsay et al., 2003). A process map visually portrays the systematic flow of sub-processes from start to finish ( Wilson, 2004). According to ( Sinclair & Zairi, 1995a, 1995b) performance measures are classified into inputs, processes, and outputs. The NDEA-based PMS defines input and output factors for each subprocess involved in the production line, enabling efficiency evaluation, benchmarking, and process improvement.

Regular assessments of the production system’s performance—daily, weekly, or monthly—are crucial during the implementation phase. Data collection precedes the calculation of efficiency scores using NDEA software. The model outcomes provide performance scores for each DMU, identifying top performers. For underperforming DMUs, the NDEA model pinpoints sub-processes causing inefficiencies.

In the review phase, two scenarios arise: for major modifications affecting the entire manufacturing process, the cycle resets to the start of the framework. For minor process changes, revisiting the last two stages—implementation and review—is sufficient. Minor alterations involve reviewing improvement targets and benchmarking against peer groups to align with the best performers.

## Results and discussion

The study evaluated the performance of the IV-set production line using five NDEA models, ranging from single-stage to six-stage configurations. Key findings from the analysis include:

• **Discrimination Power of NDEA Models:** The analysis revealed that while increasing the number of stages provides greater granularity and insights into specific processes, it reduces the discrimination power of the model. The six-stage model, for instance, classified a disproportionately high number of Decision-Making Units (DMUs) as efficient, limiting its utility for pinpointing inefficiencies. In contrast, the three-stage model offered a balance between granularity and discrimination power, making it the most practical choice for performance evaluation.
• **Efficiency Scores and Peer Groups:** Using the three-stage model, half of the production months (DMUs) were classified as inefficient, providing actionable insights for process improvement. Inefficient DMUs were benchmarked against peer groups, which served as reference points for achieving higher efficiency. For example, DMU 02_23 was benchmarked against 07_23 and 11_23, identifying specific targets for reducing material waste and machine downtime.
• **Stage-Specific Insights:** Analysis of individual stages revealed that the PVC granulation stage (WS1) was the least efficient and exhibited the highest performance variability. In contrast, the assembly stage (WS5) showed the highest average efficiency and the least variability, highlighting its stabilizing role in overall production.
• **Development of Practical Framework:** The study also led to the development of a practical framework for implementing NDEA-based PMS in manufacturing settings. This framework provides structured guidance for managers on stage selection and process consolidation to enhance discrimination power without sacrificing granularity. It emphasizes the importance of benchmarking and continuous monitoring to drive process improvements and efficiency gains.

The study’s results highlighted some practical implications, as follows:

▪ Strategic stage selection is crucial, with managers advised to balance stage design to capture key interdependencies without compromising discrimination power and group processes with minimal variability.
▪ By adopting the NDEA-based PMS, managers can focus on the most inefficient stages and their sources of inefficiency. For example, in the IV-set production line, optimizing machine schedules and improving raw material quality in WS1 could significantly enhance overall efficiency.
▪ The proposed NDEA-based PMS framework is adaptable to other industries with complex, multi-stage production systems, such as other industries with complex, multi-stage production systems, like automotive or electronics, to identify bottlenecks and improve throughput.
▪ The NDEA-based PMS allows data-driven decision-making. Hence **,** regularly updating efficiency scores and monitoring peer groups can help managers identify emerging inefficiencies and adapt processes accordingly.

## Conclusion

This paper contributes to the manufacturing PMS research domain in several ways. First, it initiates the integration of NDEA into PMS for a manufacturing process to develop a practical framework termed “NDEA-based PMS”. Second, the case study that investigated the application of NDEA in a pharmaceutical production line shed light on the intricacies of the shop floor by modelling the performance indicators for a multi-stage production line and highlighting the relevance of NDEA in manufacturing performance measurement and process improvement. The practical framework proposed from the insights of the case study, has answered RQ1, expanding the application of NDEA, highlighting its ability to decompose production stages and providing insightful information for strategic decision-making.

In addition, this study adds to the NDEA stream by revisiting ( Castelli et al., 2010), who asserted increased discrimination power under the NDEA model when compared to the classical model. Interestingly, the findings presented in this study contradict those of the classical DEA, which demonstrated greater discrimination power and better distinction of efficiency scores for DMUs. The exploration of the five NDEA models in this case study arrives at the conclusion that increment in stages within the NDEA model diminishes discrimination power (RQ2).

Despite all its contributions, the study is not without limitations. The proposed NDEA-based PMS framework, which was implemented in the context of pharmaceutical production lines, demands further validation for generalization across industries after considering the vast variations in manufacturing settings and processes. Data availability and quality, assumed homogeneity within production units, static modelling, and the assumption of linear processes present challenges that should be resolved to promote broader applicability.

Future research should focus on external validation of the NDEA-based PMS framework across diverse manufacturing contexts and industries to enhance generalizability. Researchers may implement various methods to investigate the weights and relationships between performance measures. Exploring the dynamics of the manufacturing system over time is a promising avenue. Dynamic NDEA models may capture changes in efficiency, process interactions, and improvement targets to offer a more comprehensive view of the evolving manufacturing landscape.

In conclusion, this research bridges a significant gap in the literature by integrating NDEA into a practical PMS framework, addressing the unique challenges of multi-stage manufacturing systems. The model’s capacity to grant detailed visibility and flexibility qualifies it as a transformative tool for researchers and practitioners. Future research is advised to persist in aligning operational practice with strategic intent and continue to serve the NDEA-based PMS framework as a portal to long-term competitiveness and efficiency in the complicated and globalized manufacturing landscape.

## Ethics statement

This study did not involve human participants, human tissue, or personally identifiable information. The analysis was conducted using anonymized internal production data obtained from a private manufacturing firm under a confidentiality agreement. As such, the study falls outside the scope of research involving human subjects as defined by the Declaration of Helsinki, and formal approval by an Institutional Review Board (IRB) or ethics committee was not required.

Nevertheless, the research protocol and data use were reviewed and approved by the Research Ethics Committee of the author’s institution to ensure compliance with institutional ethical standards and data governance requirements. No permit or reference number was issued, as the study did not involve human subjects or data requiring formal ethical clearance.

## Data Availability

The dataset used in this study comprises confidential internal production data obtained from a private manufacturing firm. Due to the sensitive nature of the data and the confidentiality agreement in place, the dataset cannot be made publicly available. Sharing the data would risk disclosing proprietary information and commercially sensitive operational details. This research involved no human subjects, and therefore did not require formal Institutional Review Board (IRB) approval. However, the data access and use were reviewed and approved by the research team’s affiliated institution to ensure compliance with ethical and contractual obligations. Researchers interested in accessing the dataset for verification or replication purposes may submit a formal request to the corresponding author. Access may be granted under specific conditions, including the signing of a non-disclosure agreement (NDA) and written approval from the data provider. All such requests will be evaluated on a case-by-case basis in accordance with the data provider’s confidentiality policies.
