# Enhancing Efficiency in Multi-Stage Pharmaceutical Manufacturing: A Process-Based Network DEA Approach

**DOI:** 10.12688/f1000research.166387.2

**Published:** 2025-12-04

**Authors:** Syarifa Hanoum, Mahmood Shubbak

**Affiliations:** 1 Department of Business Management, Institut Teknologi Sepuluh Nopember, Surabaya, 60111, Indonesia; 2 Department of Management, College of Economics and Political Science, Sultan Qaboos University, Muscat, Muscat Governorate, 123, Oman

**Keywords:** Network Data Envelopment Analysis, NDEA, Performance Measurement, Efficiency, Process Improvement, Decision-Making, Pharmaceutical Industry.

## Abstract

**Background:**

Manufacturing inefficiencies result in substantial financial losses for global industries. The present study introduces a robust Performance Measurement System (PMS) incorporating Network Data Envelopment Analysis (NDEA) to address efficiency challenges in multi-stage manufacturing systems.

**Methods:**

The study employs a case study approach within the pharmaceutical industry to reveal the pragmatic application of NDEA, which serves as the primary analytical instrument for evaluating performance across diverse production stages. Focusing on the production processes of intravenous (IV) sets, the research aims to highlight how NDEA disaggregates interconnected processes and quantify efficiency measures to pinpoint sources of inefficiencies in particular production stages and actionable insights for operational improvement. The analysis also explores the trade-off between model complexity and discrimination power as the number of stages increases.

**Results:**

First, the NDEA-based PMS provides insights to address specific process inefficiencies on the shop floor, providing strategic insights for process improvement. Second, despite its power in pinpointing the source of inefficiency, modelling a process-based PMS faces a challenge as increasing the number of stages in the model presents a trade-off between the accuracy and discrimination power of the NDEA model.

**Conclusions:**

This study holds significance within the broader field of performance measurement and efficiency analysis by bridging theoretical modelling and practical implementation. It advances existing knowledge through the integration of NDEA into a process-based PMS, offering a novel analytical framework for multi-stage manufacturing systems. By examining the trade-off between model complexity and discrimination power, this research contributes new methodological insights and extends the applicability of NDEA in real-world industrial settings. The framework offers managers actionable guidance for optimizing multi-stage manufacturing operations and contributes novel insights into the methodological behaviour of NDEA. Ultimately, this work strengthens the linkage between performance measurement theory and industrial practice, positioning NDEA as a valuable tool for continuous improvement in manufacturing systems.

## Introduction

In today’s rapidly evolving business landscape, the fierce competition for innovative products and services necessitates a focus on uniqueness to achieve success in both domestic and international markets ( Aziz et al., 2022; Handri et al., 2021, Shubbak, 2018). As companies strive to differentiate themselves, they face the daunting reality that manufacturing inefficiencies drain billions of dollars from global industries annually. This financial burden underscores the pressing need for robust performance measurement tools, which are crucial for enhancing competitiveness and fostering the seeds of innovation necessary for future success ( Mio et al., 2022). Performance measurement is a vital process for organizations seeking to evaluate efficiency and effectiveness, aligning operational activities with strategic goals ( Dahinine et al., 2024; Melnyk et al., 2014; Neely et al., 2002; Pujotomo et al., 2022). Robust and flexible performance measurement tools are not just administrative necessities, they are strategic enablers that help organizations navigate financial burdens, enhance competitiveness, and foster the innovation and market dynamics needed for future success ( Calik, 2024; Xing et al., 2025; Xu & Zhu, 2024).

In the manufacturing sector, where process efficiency is critical, Performance Measurement Systems (PMS) are pivotal in tracking, evaluating, and enhancing performance, to ensure the production process is in a cost-effective manner ( Hanoum, 2021; Hanoum & Islam, 2021; Rodríguez et al., 2024). Manufacturing firms should go above and beyond to achieve their strategic goals and increase their performance ( Azizi et al., 2025a, b; Handri et al., 2021).

Despite their recognized significance, current PMS frameworks frequently fail to deliver practical guidance for operationalizing performance indicators at the process level. Established models, such as the Balanced Scorecard ( Kaplan & Norton, 1996), the Baldrige Excellence Framework (BEF) ( Arif, 2007), and the European Foundation for Quality Management (EFQM) model ( Doeleman et al., 2014), highlight strategic alignment but often lack comprehensive methodologies for implementing performance measures in complex, multi-stage manufacturing contexts ( Neely et al., 2002; Van Looy & Shafagatova, 2016). The current PMS frameworks also lack guidance on how process performance measures are chosen and operationalized in practice.

Recent studies have highlighted that most PMS frameworks remain focused on high-level, strategic, and financial indicators, offering limited capability to capture the dynamic interactions and process-level performance inherent in complex production systems ( Ma et al., 2025; Tavassoli, 2025; S. Wu et al., 2025; Ye et al., 2025). Without integrating process-based and data-driven perspectives, organizations struggle to translate strategic objectives into actionable operational improvements. These observations underscore the pressing need for refined analytical approaches capable of systematically evaluating multi-stage operations, identifying stage-specific inefficiencies, and providing granular insights to guide process optimization. This gap underscores the need for frameworks that align with strategic objectives and provide in-depth operational processes tailored to specific environments. Additionally, it underlines the need for advanced analytical tools that quantify efficiency and deliver detailed insights into specific process-related inefficiencies.

Network Data Envelopment Analysis (NDEA) presents a robust framework by representing manufacturing operations as interlinked processes, thus capturing inputs and outputs across multiple stages of production. Unlike conventional Data Envelopment Analysis (DEA), which often treats production systems as monolithic entities or ‘black boxes’, NDEA disaggregates these systems. This enables a more granular examination of inefficiencies, allowing for the identification of targeted areas for enhancement ( Färe & Primont, 1984; Tone & Tsutsui, 2009). By integrating NDEA into a process-based PMS, organizations can bridge the gap between strategic intent and operational execution, offering a practical and analytically rigorous framework for multi-stage manufacturing environments.

While existing research has demonstrated the utility of NDEA for benchmarking and efficiency evaluation, there remains a lack of studies applying NDEA within single enterprises to develop a process-based PMS ( Castelli et al., 2010; Kao, 2014; Rachmad et al., 2024). Additionally, the impact of increasing stages on the discrimination power of NDEA models remains underexplored. Addressing these gaps, this study investigates the following research questions:

▪ The practical application of NDEA in real-world settings faces numerous challenges ( J. S. Liu et al., 2013; Paradi & Sherman, 2014). To address this, the study asks: How can a NDEA-based PMS be practically implemented to improve multi-stage manufacturing processes? **(RQ1).**
▪ Several DEA studies have analysed the internal structure of Decision-Making Units (DMUs) and found that NDEA has a stronger discrimination power compared to classical DEA ( Castelli et al., 2010). This study aims to verify and expand on this theory by posing the question: How does the number of stages in a NDEA model affect its discrimination power? **(RQ2).**

To answer these questions, this article presents a case study focused on the production line of a pharmaceutical company’s intravenous (IV) sets, exemplifying the intricacies of multi-stage manufacturing, featuring a combination of manual and automated processes. The case study is intentionally illustrative; the goal is to demonstrate the feasibility and practical utility of a NDEA-based PMS rather than to generate statistically generalizable results. Using a case study of intravenous (IV) set production, the research illustrates how NDEA can be operationalized to:

• Provide a practical framework for routine efficiency monitoring and process improvement.
• Identify stage-specific inefficiencies as the focus of process improvement.
• Evaluate trade-offs between model granularity and discrimination power.

By embedding model selection criteria, incorporating stage validation with production engineers, and accounting for undesirable outputs, the study provides a robust methodology for implementing NDEA in operational settings. In doing so, it bridges the theoretical development of network DEA with practical performance management needs in complex manufacturing environments.

The structure of the article is organized as follows: the next section provides a literature review on NDEA, tracing its evolution from DEA to NDEA, discussing fundamental concepts, and detailing the selection of NDEA models. A section on methodology follows, presenting the stages of the research. The subsequent section presents a case study that illustrates the application of NDEA within an IV sets production line, in a pharmaceutical company. Following this, we explore the proposed practical NDEA-based performance management system (PMS) derived from the case study. The concluding sections summarize the key findings, draw conclusions, and make recommendations for future research.

## Literature review: Network data envelopment analysis

### From DEA to NDEA

Data Envelopment Analysis (DEA) is a widely used methodology for evaluating efficiency across decision-making units (DMUs) based on inputs and outputs. However, its “black-box” approach often fails to capture the complexities of multi-stage systems where intermediate outputs are significant ( Färe & Primont, 1984; Seiford & Zhu, 1999). To address this limitation, Network Data Envelopment Analysis (NDEA) extends DEA by modelling the internal structure of DMUs, providing a more granular assessment of efficiency ( Tone & Tsutsui, 2009). NDEA offers unique advantages over classical DEA, including the ability to disaggregate multi-stage processes into sub-processes, enabling a detailed evaluation of inefficiencies ( Färe & Primont, 1984), and improve strategic decision-making by identifying performance bottlenecks across stages ( Kao, 2014). Table 1 summarizes key theoretical advancements of NDEA compared to DEA.

**Table 1.** The key theoretical advancements of NDEA compared to DEA.

Aspect	DEA	NDEA
System Structure	Treats DMUs as “black boxes” without internal process analysis.	Models interconnected sub-processes within DMUs.
Intermediate Outputs	Does not account for intermediate products or services.	Explicitly incorporates intermediate outputs between stages.
Application Scope	Primarily used for single-stage or static systems.	Tailored for multi-stage, dynamic manufacturing systems.

Recent literature demonstrates that the application of network-based efficiency measurement tools has expanded significantly across diverse industrial contexts, demonstrating its capability to assess multi-stage efficiency and support process-level decision-making. For example, Vogt et al. (2025) applied NDEA in a steel manufacturing company to benchmark efficiency in bar and profile rolling processes, identifying stage-specific inefficiencies and providing actionable insights for operational improvement. Additionally, NDEA has been integrated with machine learning to evaluate supply chain sustainability, demonstrating the effectiveness of hybrid approaches ( Koushki & Naghdehforoushha, 2025). Furthermore, Ahmadi et al. (2024) proposed an NDEA model capable of handling fuzzy data, offering a robust framework for measuring efficiency under uncertainty and complex production conditions. In essence, the shift from DEA to NDEA reflects a broader conceptual and practical progression: from measuring overall efficiency to understanding the dynamics of process-level performance and generating actionable insights that drive improvements. As industrial systems grow more complex and data-rich, NDEA and its adaptations will be crucial for informed, stage-specific decision-making.

### The basic concept of NDEA

**Figure 1.**
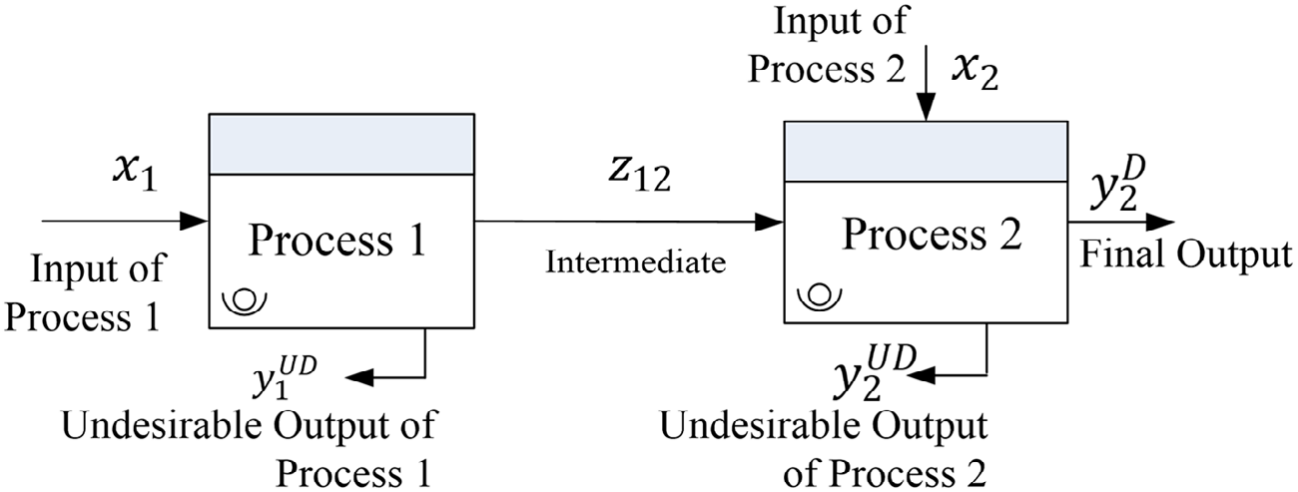
Interaction among input, output, and intermediate factors in two-stage manufacturing operations. The figure illustrates the flow of desirable and undesirable outputs, including intermediate products (z12) between two processes, and the role of inputs (x1, x2) and final output (y2).

Färe and Primont (1984) initiated the exploration of the ‘black-box’ system of classical DEA and was followed by other scholars ( Seiford & Zhu, 1999; Wang et al., 1997). Figure 1 illustrates the interactions among inputs (x _i_), outputs (y _r_), and intermediate factors (z _kh_) in two-stage manufacturing operations. S _(k,h)_ represents the number of intermediate measures passing from the k ^th^ process to the h ^th^ process. Process 1 has input (x _1_) and the output of process 1, which is called intermediate output (z _12_), becomes the input in process 2. In addition to z _12_, process 2 has a supplementary input (x _2_) that yields an output (y _2_). Given that process 2 is the final process, y _2_ is the final output of the manufacturing operations.

While the intermediate and final products are characterized as desirable outputs, a manufacturing process often generates undesirable outputs including rejected products and waste. Figure 1 presents the undesirable outputs produced by both processes (y _1_^UD^ and y _2_^UD^). Given that desirable outputs are expected to be maximized, undesirable outputs must be minimized. Some authors have incorporated undesirable outputs in the DEA model ( Chung et al., 1997; Scheel, 2001).

### Selections of the NDEA models

(a) Distance, orientation, and scale assumptions

Researchers may choose between the radial and non-radial NDEA models. The radial approach works under the proportionality assumption in the changes of inputs or outputs. Because in manufacturing operations, production factors such as labour, materials, and capital may not be changed proportionally, we choose the network slacks-based measure (NSBM) that operates the efficiency measurement based on input excess and output shortfall ( Tone & Tsutsui, 2009). The choice of orientation between input and output depends on whether the decision-makers’ focus is on controlling inputs or outputs to improve efficiency. A non-oriented approach, as adopted here, avoids subjective prioritization and allows the model to identify which inputs or outputs require improvement purely based on data. Scale assumptions are also critical ( Zuniga-Gonzalez et al., 2025). While variable returns to scale (VRS) accommodates differing economies of scale among decision-making units, constant returns to scale (CRS) are appropriate when evaluating operations within a single plant where scale effects are minimal.

In DEA literature, the combination of non-orientation, non-radial, and VRS modelling is often used to enhance the relevance of frontier efficiency studies ( Avkiran, 2011). VRS is often highlighted for accommodating the differing economies of scale among Decision-Making Units (DMUs), where scale is not constant in nature ( Zuniga-Gonzalez et al., 2025). However, this study evaluates a manufacturing line across various production periods where economies of scale are not a concern. Aparicio & Santín (2025) further refines this understanding through the concept of global scale efficiency, emphasizing that CRS assumptions are justified when scale variation is not structurally embedded in the production system. Given these factors, the choice of a non-oriented, non-radial, CRS-NSBM is methodologically justified for multi-stage manufacturing operations within a single plant. This model effectively captures non-proportional slack adjustments, accommodates both desirable and undesirable outputs, and eliminates scale distortions, thereby providing reliable and actionable insights into inefficiencies specific to each stage of production.

(b) The presence of undesirable outputs

NDEA models maximise the outputs of a system to achieve the system’s optimum efficiency. Manufacturing environments, however, may generate undesirable outputs such as production wastes, rejected products, and pollution. Therefore, NDEA, which incorporates undesirable outputs in its mathematical model, is considered. We follow ( W. B. Liu et al., 2010) by applying this undesirability phenomenon to the NSBM approach given by Tone and Tsutsui (2009) as presented in Model 1. The extended model is characterised as NSBM-non-oriented-CRS with undesirable outputs.

$$\begin{array}{c} \mathrm{Min}\;\rho_{o}^{*}=\frac{\sum_{k=1}^{K}W^{k}\left[1-\frac{1}{m_{k}}\left(\sum_{i=1}^{m_{k}}\frac{s_{i}^{k-}}{x_{\mathrm{io}}^{k}}\right)\right]}{\sum_{k=1}^{K}W^{k}\left[1+\frac{1}{R_{k}^{D}+R_{k}^{\mathrm{UD}}}\left(\left(\sum_{r_{k}^{D}=1}^{R_{k}^{D}}\frac{S_{r}^{k{+}^{D}}}{y_{\mathrm{ro}}^{k^{D}}}\right)+\left(\sum_{r_{k}^{\mathrm{UD}}=1}^{R_{k}^{\mathrm{UD}}}\frac{S_{r}^{k{+}^{\mathrm{UD}}}}{y_{\mathrm{ro}}^{k^{\mathrm{UD}}}}\right)\right)\right]}\\ \text{Subject to}\\ \sum_{j=1}^{n}x_{\mathrm{ij}}^{k}\lambda_{j}^{k}-S_{i}^{k-}\leq x_{\mathrm{io}}^{k},\;i=1,\ldots ,m_{k},\;k=1,\ldots ,K\;\\ \sum_{j=1}^{n}y_{r_{k}^{D}j}^{k^{D}}\lambda_{j}^{k}-S_{r}^{k{+}^{D}}=y_{r_{k}^{D}o}^{k^{D}},\;r_{k}^{D}=1,\ldots ,R_{k}^{D},\;k=1,\ldots ,K\\ \sum_{j=1}^{n}y_{r_{k}^{\mathrm{UD}}j}^{k^{\mathrm{UD}}}\lambda_{j}^{k}-S_{r}^{k{+}^{\mathrm{UD}}}=y_{r_{k}^{\mathrm{UD}}o}^{k^{\mathrm{UD}}},\;r_{k}^{\mathrm{UD}}=1,\ldots ,R_{k}^{\mathrm{UD}},\;k=1,\ldots ,K\\ \sum_{j=1}^{n}\sum_{s_{\left(k,h\right)}=1}^{S_{\left(k,h\right)}}z_{s_{\left(k,h\right)}j}^{\left(k,h\right)}\lambda_{j}^{k}=\sum_{j=1}^{n}\sum_{s_{\left(k,h\right)}=1}^{S_{\left(k,h\right)}}z_{s_{\left(k,h\right)}j}^{\left(k,h\right)}\lambda_{j}^{h},\;\forall \left(k,h\right),\;\lambda_{j}^{k},S_{r}^{k+}\geq 0 \end{array}$$ (1)

The study’s sample size would be n DMUs (j = 1, …, n), which consist of K sub-processes (k = 1, …, K). The objective $\rho_{o}^{*}$ characterises the non-oriented efficiency score of DMUo (the subscript “o” represents the DMU under analysis), while $w^{k}$ in this function is denoted as a subjective weight of the kth sub-process. Because the subjectivity is avoided in this study’s decision-making process, the weights are set as 1.00 for all sub-processes. If $\rho_{o}^{*}=1$ and all input and output slacks are equal to zero, then the DMUo is efficient. The method decomposes the overall efficiency into divisional/process efficiency ( $\rho_{k}$ ), given in model (2), where $s_{i}^{k-*}$ and $s_{r}^{k+*}\;$ are the optimal input-slack and output-slack for model (1). All nomenclatures of models (1) and (2) are described in Table 2.

$$\rho_{k}=\frac{1-\frac{1}{m_{k}}\left(\sum_{i=1}^{m_{k}}\frac{s_{i}^{k-*}}{x_{\mathrm{io}}^{k}}\right)}{1+\frac{1}{r_{k}}\left(\sum_{r=1}^{r_{k}}\frac{s_{r}^{k+*}}{y_{\mathrm{ro}}^{k}}\right)},\;k=1,\ldots ,K\;$$ (2)

**Table 2.** The nomenclature of model 1.

Subscript “ *o*” is related to the DMU which is under observation		
$\rho_{o}^{*}$	=	The overall non-oriented efficiency score of DMU _o_.
$w^{k}$	=	The weight of the *k*th process/division determined by decision-makers.
$x_{\mathrm{ij}}^{k}$	=	The *i* ^th^ input, *i* = 1, …, m _k_, which corresponds to the *k* ^th^ ( *k* = 1, …, *K*) process of the *j* ^th^ DMU ( *j* = 1, …, *n*).
$y_{\mathrm{rj}}^{k}$	=	The *r* ^th^ output, *r*= 1, …, r _k,_ which corresponds to the *k* ^th^ ( *k* = 1, …, *K*) process of the *j* ^th^ DMU ( *j* = 1, …, *n*).
$S_{r}^{k+}$	=	Amount of slack related to the *r* ^th^ output of the *k* ^th^ ( *k* = 1, …, *K*) process.
$z_{s_{\left(k,h\right)}j}^{\left(k,h\right)}$	=	An intermediate factor from the *k* ^th^ process to the *h* ^th^ process ( *k* ≠ *h* and *k*, *h* = 1, …, K).
$\lambda_{j}^{k}$	=	The intensity vector corresponding to the *k* ^th^ process of the *j* ^th^ DMU.
*n*	=	Number of DMUs.
*K*	=	Number of processes/divisions.
$m_{k}$	=	Number of inputs corresponding to the *k* ^th^ process.
$r_{k}^{D}$	=	The subscript corresponding to desirable outputs of the divisions/processes.
$R_{k}^{D}$	=	Number of desirable outputs corresponding to the *k* ^th^ process.
$r_{k}^{\mathrm{UD}}$	=	The subscript corresponding to undesirable outputs of the divisions/processes.
$R_{k}^{\mathrm{UD}}$	=	Number of undesirable outputs corresponding to the *k* ^th^ process.
$s_{\left(k,h\right)}$	=	The subscript of intermediate measure from the *k* ^th^ process to *h* ^th^ process.
$S_{\left(k,h\right)}$	=	Number of intermediate measures passing from the *k* ^th^ process to *h* ^th^ process.
$\rho_{k}$	=	The divisional/process efficiency of the *k* ^th^ process.

Recent research has advanced the treatment of intermediate and undesirable outputs, refining NDEA models to better capture complex network structures, dual-role factors, and process interdependencies. For example, Lotfi et al. (2023) applied an NDEA model to assess both desirable and undesirable outputs in the wheat supply chain, demonstrating enhanced accuracy in identifying stage-specific inefficiencies. Ma et al. (2025) proposed a network slack-based measure incorporating dual-role factors and undesirable outputs to evaluate supply chain performance, highlighting the practical relevance of NDEA in complex production networks. Yang et al. (2024) further demonstrate the flexibility of NDEA by incorporating shared resources, negative data, and undesirable outputs in a multi-stage airline efficiency context, highlighting its capacity to model interdependencies realistically. This development allows decision-makers to simultaneously optimize performance while reducing waste or other negative byproducts, thereby providing a more nuanced and actionable understanding of operational efficiency. Essentially, NDEA’s ability to model undesirable outputs transforms efficiency assessment into a more realistic and strategically valuable instrument for complex production systems.

## Methodology

The research begins with a comprehensive literature review to identify the most appropriate Non-Directional Efficiency Analysis (NDEA) model for multi-stage manufacturing. The review covered radial and non-radial approaches, constant and variable returns to scale (CRS/VRS), and input-, output-, and non-oriented structures. Building on this synthesis, we adopted the combined non-radial Non-SBM (NSBM) non-oriented CRS model as the analytical foundation for this study, as it is well-suited for capturing slacks, handling undesirable outputs, and modelling internal product flows within a single plant.

The study uses a specific criterion for selecting the preferred network model: it should minimize the number of efficient DMUs while capturing key stages of production. This approach balances simplicity and adequacy, enabling systematic comparisons of network configurations rather than relying on qualitative judgments. This criterion ensures that the desired structure balances parsimony with adequacy, and provides a systematic basis for comparing alternative network decompositions rather than relying solely on qualitative judgment.

To pinpoint the optimal number of stages for our pharmaceutical production line, we explored network configurations ranging from 2 to a comprehensive 4-stage model. This intricate design process was undertaken in close collaboration with both the production manager and the supervisory team, ensuring that each stage was thoughtfully validated. Together, we analysed the state groupings, carefully considering how integrating specific stages could optimise efficiency and streamline our operations. The discussions were rich and detailed, reflecting our commitment to creating a production network that not only meets our standards but also enhances the overall workflow.

The next phase of the research involved an in-depth case study in which the selected NDEA model and three-stage structure were applied to a pharmaceutical manufacturing system. It is important to note that this case study serves as an illustrative example to demonstrate the practical implementation of the proposed NDEA-based PMS. Due to the single-case design, statistical validation or generalization of the results is not applied, and the findings primarily highlight methodological feasibility and operational insights rather than inferential conclusions.

The case study application enabled us to identify the practical challenges of implementing NDEA within a performance measurement system (PMS), including data collection, stage decomposition, and the handling of undesirable outputs. The case study results informed a set of managerial recommendations, specifying sources of inefficiency and stage-specific improvement targets across the production line.

Drawing from these findings, we developed a practical framework—referred to as the NDEA-based PMS—to guide managers in applying NDEA in multi-stage manufacturing settings. This framework synthesizes methodological rigor, empirical insights, and practitioner expertise, offering a structured approach to designing performance indicators, diagnosing inefficiencies, and supporting continuous process improvement within complex manufacturing environments.

### Applying NDEA to measure performance of a pharmaceutical production process: A case study

This case study illustrates the transformation of manufacturing operations through the application of the NDEA model. It explores the practical implementation of NDEA to establish a multi-stage Performance Measurement System (PMS) within a pharmaceutical production environment. The study aims to achieve two main objectives: developing a practical, process-based performance measurement framework for single-enterprise, multi-stage systems, and examining the trade-off between NDEA model complexity and discrimination power. The case study is illustrative in nature, and due to the single-case design, statistical validation is not applied.

The pharmaceutical company occupies approximately 60,000 square meters. It promotes four product groups: intravenous sets (IV Sets), IV Solutions, Therapeutic Drugs, and Clinical Nutrition. The company dominates the market with a 70% share in basic solutions and medicines, catering to both domestic and international markets. Its operations adhere to high regulatory standards and stringent quality requirements, making efficiency and waste minimization crucial for maintaining competitiveness.

This study focuses on the production line for IV sets, medical devices used to deliver intravenous fluids or perform blood transfusions. The IV set line was chosen for this study because it represents one of the highest-revenue product lines. This strategic selection is based on several important reasons:

▪ **Strategic Importance:** IV sets represent one of the company’s highest-revenue product lines, accounting for nearly 30% of total sales. Optimal performance in this line directly impacts overall profitability.
▪ **Operational Complexity:** The production of IV sets involves combination of machining and manual processes, which present operational challenges for executives when managing performance fluctuations.
▪ **Process Variability:** The IV-set line frequently experiences performance fluctuations due to machine downtime, material inconsistencies, and manual assembly errors. These challenges underscore the need for a robust performance measurement framework to identify inefficiencies and guide process improvements.
▪ **Scalability and Relevance:** Insights from the IV-set line can be generalized to other product lines within the company, as many share similar multi-stage production structures.

The IV production line experiences significant performance fluctuations, with early analysis revealing concerning trends: PVC granulation waste rates of 30%-35%, indicating substantial material loss. Additionally, machine downtime is a persistent issue, accounting for 15% to 20% of scheduled production hours, disrupting workflow and hampering overall efficiency. Furthermore, the output from manual assembly processes shows significant variability, largely influenced by the skill and consistency of the operators involved. This mix of inefficiencies underscores the need for targeted improvements to boost productivity and reduce waste.

The study examines a 12-month production period, chosen to capture operational variability across the full production cycle, including seasonal fluctuations in raw material supply, maintenance schedules, and staffing. The monthly aggregation aligns with the company’s internal reporting and corporate board review processes, providing both sufficient granularity and reliability for performance evaluation.

Production engineers and line supervisors actively contributed to this study. Their participation included validating the NDEA model structure, confirming stage groupings, and reviewing the selected inputs, outputs, and intermediate variables for each production stage. While the NDEA analysis provided actionable insights and was shared with managers, it was not formally implemented in daily operational control during the study period. Nevertheless, preliminary discussions were initiated within the company to consider targeted improvements, such as reducing PVC granulation waste and optimizing machine schedules. These steps demonstrate the potential practical utility of the proposed NDEA-based PMS for guiding future process improvements.

### Process mapping

**Figure 2.**
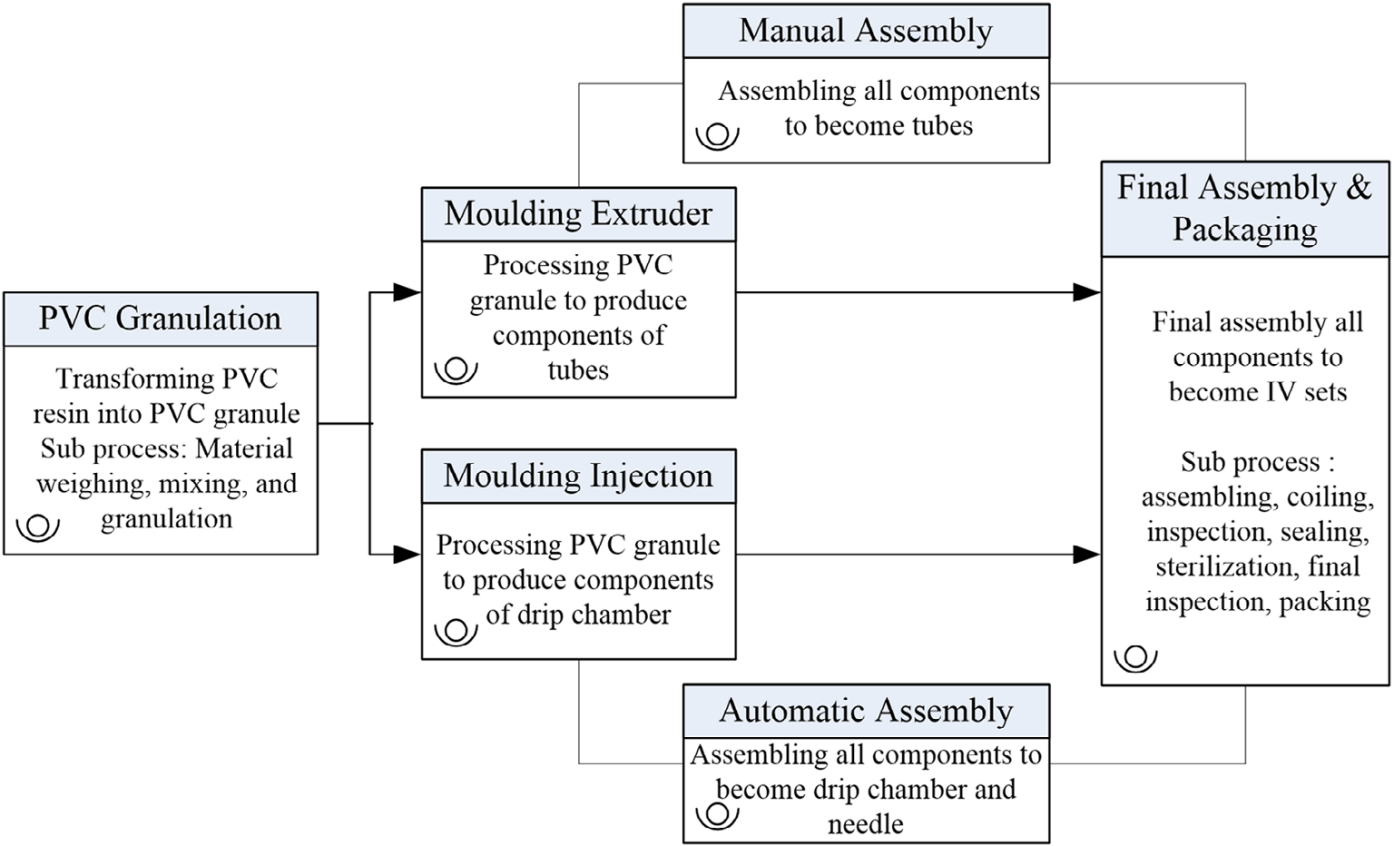
Process map of the intravenous (IV) set production process. This diagram outlines six interconnected workstations: PVC Granulation, Moulding Extruder, Moulding Injection, Manual Assembly, Automatic Assembly, and Final Assembly & Packaging.

IV-sets are produced across six interconnected workstations, as depicted in Figure 2. These stages include:

**PVC Granulation (WS1):** Conversion of raw Polyvinyl Chloride (PVC) resins into medical-grade granules.

▪ **Moulding Extruder (WS2):** Fabrication of sub-assembled tubes using the granulated PVC.
▪ **Moulding Injection (WS3):** Production of components such as drip chambers, spikes, and needle covers.
▪ **Manual Assembly (WS4):** Manual combination of sub-assembled tubes and additional components.
▪ **Automatic Assembly (WS5):** Automated assembly of finished IV-set components, including drip chambers.
▪ **Final Assembly and Packaging (WS6):** Integration of all components, final quality checks, and packaging.

Each stage is interdependent, with outputs from one stage serving as inputs for the next. This multi-stage structure makes the IV-set line an ideal case for applying NDEA, which accounts for intermediate outputs and interconnected processes.

### NDEA variables and model specification

In NDEA studies, model specification is crucial to list the performance assessment criteria. However, studies that have rationalized the essential variables for performance assessment are scarce ( Cook et al., 2014; Kishore et al., 2024). Once the process map is available, identifying inputs and outputs for each process becomes more straightforward when adopting the NDEA for manufacturing operations.

The initial process involves consuming various PVC resins as raw materials to produce non-toxic medical-grade PVC granules. These granules serve as inputs for the subsequent moulding processes. In the second stage, moulding involves the addition of additional materials to create a range of components. The moulding extruder generates an assortment of sub-assembled tubes, whereas the moulding injection yields drip chamber components (e.g., the central part of drip chambers, spikes, needle covers, joints, and seals) for the following assembly process. Both PVC granulation and moulding processes require operators and machines.

The assembly processes combine all the parts produced by the prior workstations. The assembly station comprises three sub-stations: automatic assembly, manual assembly, and final assembly. The automatic assembly combines components from moulding injection into the finished drip chamber unit. It is characterized as a one-man-one-machine workstation, where machine-hour or man-hour is used interchangeably. At the manual assembly workstation, sub-assembled tubes from the moulding extruder and additional components from suppliers are manually assembled, requiring only man-hours as input. The final assembly line involves a combination of machine and manual work, which is reflected in man-hours and machine-hours.

Outputs from earlier stages, that serve as inputs to subsequent stages, are called intermediate factors. The last stage generates the final outputs. The ultimate product of this production line is the IV-Set, available in various sizes intended for the administration of nutrition, medication, and blood. In addition, the IV-Set production line yields waste or rejected outputs known as undesirable outputs. Another undesirable output refers to machine downtime that occurs in the PVC granulation, moulding extruder, and moulding injection workstations. Meanwhile, the assembling activities that utilize machines and equipment with lower breakdown risks render machine downtime - an insignificant factor in all assembly processes.

A favourable DEA/NDEA model is typically characterized by its discrimination power. The discrimination power of a DEA model can be compromised when a massive number of inputs and outputs are used, mainly because a particular number of DMUs is considered efficient in specific scenarios ( Cook et al., 2014). As a rule of thumb, the number of DMUs should be at least twice the total number of inputs and outputs. Adhering to this guideline minimizes the correlation between variables and DEA/NDEA outputs, thereby enhancing the discriminating power of the model.

**Figure 3.**
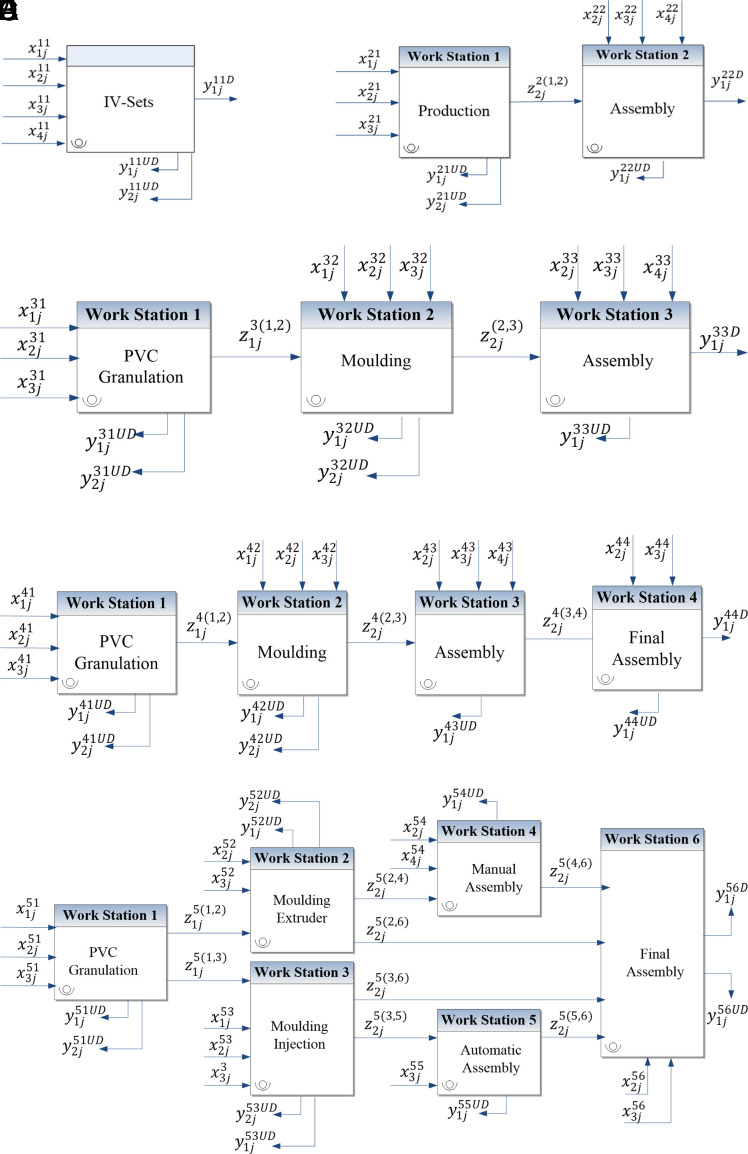
Interaction of inputs, intermediate factors, and outputs in five NDEA models. The five subfigures represent different decomposition levels of the production system: (A) classical DEA, (B) two-stage NDEA, (C) three-stage NDEA, (D) four-stage NDEA, and (E) six-stage NDEA.

According to Castelli et al. (2010), discrimination power is higher in the NDEA model, compared to the classical model. In certain cases, it is possible for most or even all DMUs to be deemed inefficient. However, the idea that adding more stages to the NDEA model improves discrimination power is inconclusive. The existing literature does not provide clear guidance on how to divide a system into multi-stage and interconnected sub-systems, nor does it specify the optimal number of stages needed for particular manufacturing operations. To bridge these gaps, this paper outlines five scenarios aimed at identifying the most appropriate NDEA model for the IV-Set production system (refer to Figure 3). The nomenclature related to Figure 3 can be found in Table 3.

**Table 3.** The NDEA data corresponding to Figure 3.

Variables	Definitions	Unit of measures
$x_{1j}^{\mathrm{lk}}$	Raw materials corresponding to the *k* ^th^ process of the *j* ^th^ DMU.	Kilograms (kgs)
$x_{2j}^{\mathrm{lk}}$	Man-hour corresponding to the *k* ^th^ process of the *j* ^th^ DMU.	Hours (hrs)
$x_{3j}^{\mathrm{lk}}$	Machine-hour corresponding to the *k* ^th^ process of the *j* ^th^ DMU.	hrs
$x_{4j}^{\mathrm{lk}}$	Additional components corresponding to the *k* ^th^ process of the *j* ^th^ DMU.	Pieces (pcs)
$y_{1j}^{\text{lkUD}}$	Rejected outputs (undesirable output) corresponding to the *k* ^th^ process of the *j* ^th^ DMU.	kgs
$y_{2j}^{\text{lkUD}}$	Machine downtime (undesirable output) corresponding to the *k* ^th^ process of the *j* ^th^ DMU.	hrs
$z_{1j}^{l\left(k,h\right)}$	Intermediate outputs from the *k* ^th^ process to the *h* ^th^ process ( *k* ≠ *h* and *k*, *h* = 1, …, K).	kgs
$z_{2j}^{l\left(k,h\right)}$	Intermediate outputs from the *k* ^th^ process to the *h* ^th^ process ( *k* ≠ *h* and *k*, *h* = 1, …, K).	pcs
$y_{1j}^{\mathrm{lkD}}$	Final output (desirable output) corresponding to the *k* ^th^ process of the *j* ^th^ DMU.	pcs
*L*	Numerator index corresponding to the NDEA model/scenario under evaluation ( *l* = 1, …, 5).	

Referring to Figure 3, Model (1) illustrates the classical DEA, where the production line is a ‘black-box’ system and the intermediate factors are dismissed. Model (2) shows the simplest form of NDEA (two-stage NDEA), where the manufacturing process of IV-Set is divided into production and assembly stages. In model (2), the intermediate factors flow between the production and the assembly stages.

Model (3) expands on Model (2) by dissecting the production stage into PVC granulation and moulding after considering the intermediate factors and undesirable outputs that flow between the three stages. Unlike Model (2), Model (3) treats the additional materials required for moulding as a separate variable. Model (4) segregates the production system into four stages based on the responsibilities of the four supervisors in the IV-Set department. Each supervisor oversees one of the following four units: PVC granulation, moulding, assembly, and final assembly.

Model (5) incorporates the six workstations outlined in the process map. It looks into the internal structure of all processes, which consist of the largest number of stages and NDEA variables. The technical correctness of each model was validated by conducting a comprehensive review by the research team and production supervisors. The focus of this validation is to ascertain that there was no significant process change throughout the one-year study period to maintain the high acceptability of the models.

### Data collection and model selection

The following sub-section details the data source, the selection of five models, and the decision support system facilitated by NDEA for performance evaluation and process improvement. The data from 12 months, including inputs, outputs, and intermediate variables, were extracted from the company. The one-year monthly production period served as the DMUs to meet the requirements of the annual review conducted by the corporate board.

The NDEA efficiency scores were computed for each production month, hence treating the IV-Set production system as a one-, two-, three-, four-, or six-stage production process. The objective is to identify a model that aligns with NDEA requirements and serves as a decision support system for process improvement. The models must exhibit acceptable discrimination power and provide accurate insights for process improvement. The basic descriptive statistics shown at the bottom of Table 4 demonstrate how effectively the models differentiated among the DMUs’ efficiency levels.

**Table 4.** Efficiency scores for NDEA model scenarios.

NO	DMU	1-stage	2-stage	3-stage	4-stage	6-stage
1	01_23	1	1	1	1	1
2	02_23	0.376	0.526	0.518	0.571	1
3	03_23	0.173	0.228	0.290	0.289	0.292
4	04_23	0.136	1	1	1	1
5	05_23	0.180	0.324	0.313	0.325	0.343
6	06_23	0.383	0.411	0.443	0.459	0.418
7	07_23	1	1	1	1	1
8	08_23	1	1	1	1	1
9	09_23	0.590	0.693	0.642	0.613	1
10	10_23	1	1	1	1	1
11	11_23	1	1	1	1	1
12	12_23	0.323	0.343	0.469	1	1
Average efficiency score		0.597	0.710	0.723	0.771	0.838
Least efficiency score		0.136	0.228	0.290	0.289	0.292
Standard deviation		0.347	0.306	0.294	0.291	0.295
Number of efficient DMUs		5	6	6	7	9

Apart from assessing whether an increased number of stages in NDEA can enhance discrimination power, examining the five models determined the most suitable model for the IV-Set production line. The discrimination power of NDEA was assessed using the following statistics: average efficiency score, minimum efficiency score, number of efficient DMUs, and standard deviation, with lower values indicating greater discrimination power.

The descriptive statistics revealed that Model (1) performed best in distinguishing the efficiency scores throughout the production period. However, selecting a single-stage DEA model is unsuitable for a process-based PMS that emphasizes internal processes. This approach provided insufficient information to uncover the specific stages and production network that demands improvements. Model (5) was the favoured model for process enhancement because it offered detailed insights by breaking down the production line into more processes than the other models. However, more stages in an NDEA model introduce additional variables that may affect discrimination power. Based on Table 3, Model (5) exhibited the lowest discrimination power, as indicated by the highest average, the lowest efficiency values, the smallest standard deviation, and the highest number of efficient DMUs.

Upon comparing Models (1), (2), and (3), the statistical results disclosed that Model (3) had lower discrimination power than Models (1) and (2) for two reasons. First, Model (3) recorded a higher average efficiency score and the lowest efficiency score when compared to Models (1) and (2). Second, Model (3) had a smaller standard deviation value than the other two. Nonetheless, the oversimplification inherent in the single- and two-stage Models (1) and (2) limited their efficacy in comprehending the production system.

After considering the trade-offs, Model (3) was selected as the preferred model for several reasons. Given its moderate number of stages, Model (3) strikes a balance between the discrimination power required by NDEA and the necessary details for process improvement purposes. From the stance of PMS, Model (3) aligns with the parsimony principle, which emphasizes data collection and processing without excessive cost and time implications.

### Efficiency scores and peer groups

The NDEA non-parametric method measures efficiency by assessing each criterion measure (weighted output/input) and constructing an envelopment frontier across all measures to ascertain that the observed data points lie on or below the frontier. The three-stage NDEA model ( Figure 3C) was deployed in this case study. Scores were computed based on the 12-month production period. The efficiency scores (see Table 5) revealed that 50% of the production period fell below the production frontier. Table 5 presents the inefficient production months and their respective peer groups. A peer group refers to the efficient months with the most similar circumstances to each inefficient month concerning the input and output sets. For example, the peer group of production period 02_23 includes 07_23 and 11_23.

**Table 5.** The efficiency score of the three-stage NDEA-based PMS.

Work-stations	Efficient DMUs						Inefficient DMUs					
	01_23	04_23	07_23	08_23	10_23	11_23	02_23	03_23	05_23	06_23	09_23	12_23
Overall	1	1	1	1	1	1	0.518	0.290	0.313	0.443	0.642	0.469
PVC	1	1	1	1	1	1	0.475	0.332	0.327	0.512	0.782	0.599
Moulding	1	1	1	1	1	1	0.629	0.340	0.332	0.628	0.735	0.443
Assembly	1	1	1	1	1	1	0.665	0.395	0.471	0.394	0.612	0.674

Peer groups (efficient months)		07_23 11_23	07_23	07_23	07_23 11_23	07_23 11_23	07_23
Descriptive statistics of the production system							
	Average efficiency score	Least efficiency score	Standard Deviation				
Overall	0.723	0.290	0.303				
PVC	0.752	0.327	0.284				
Moulding	0.759	0.332	0.277				
Assembly	0.768	0.394	0.258				

The classical DEA displayed the sources of inefficiency through input and output variables. Taking a step further, the NDEA method evaluated each stage along the network to determine the production process with the most significant impact on the overall efficiency of the manufacturing operations. More insights were captured from the NDEA results, particularly by examining the descriptive statistics (see bottom of Table 4). The PVC granulation stage recorded the lowest efficiency score, which solidified its status as the most inefficient stage and a prominent contributor to the overall inefficiency of the IV-Set manufacturing line. The standard deviation of its efficiency score was the largest, translating into considerable performance fluctuations over the studied production year. On the contrary, the assembly stage displayed the highest average efficiency score and minimal performance variability, further confirming its pivotal role in bolstering the overall production efficiency.

### Process improvement

Referring to the efficiency scores, the NDEA produced slacks for each variable in the model to signify the shortfall of outputs or the excess of inputs that rendered a DMU inefficient. Besides, the NDEA offered improvement targets for each production factor (i.e., material, man-hour, and machine-hour) and output (i.e., machine downtime, rejected outputs, and good products). Improvement can manifest as a decrease in inputs and undesirable outputs or an increase in desirable outputs.

**Figure 4.**
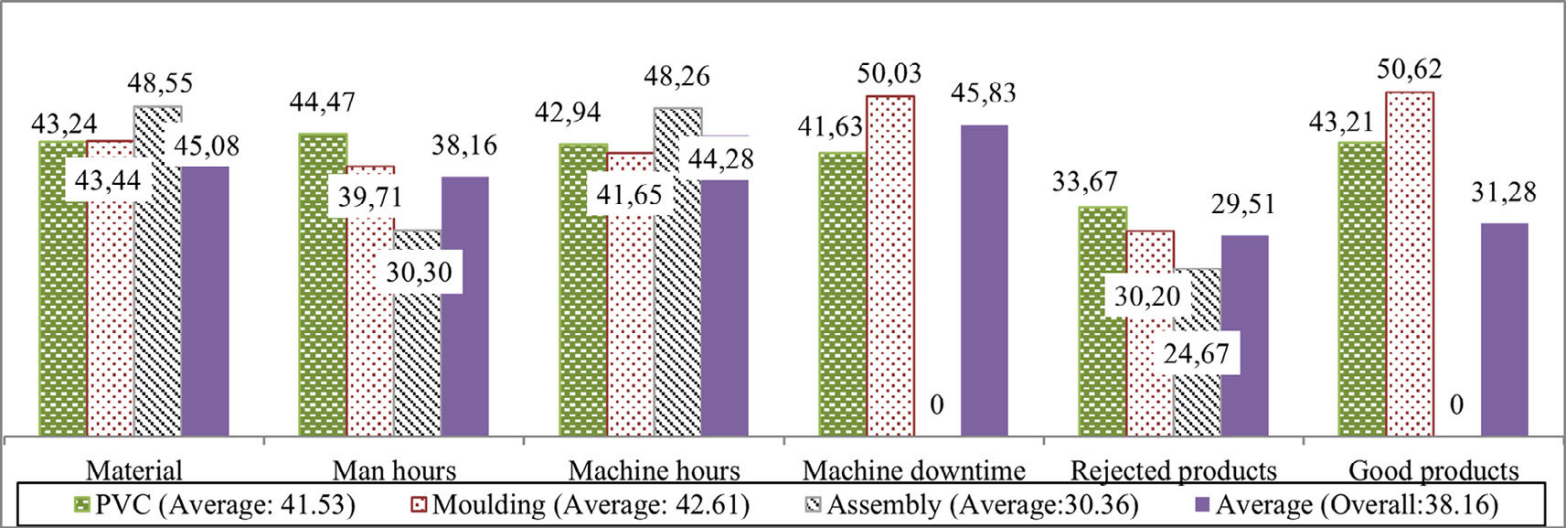
Percentage of potential improvement for the production process. This bar chart visualizes potential reductions in input materials, man-hours, machine-hours, and undesirable outputs, as well as the necessary increase in good outputs to achieve optimal efficiency.

For each category in Figure 4, the first, second, and third bars represent inputs consumed or outputs generated at the PVC granulation, moulding, and assembly workstations. The fourth bar depicts the average potential improvement required for each category.

Figure 4 illustrates the imperative need for the company to minimize input materials, man-hours, and machine-hours by 45.08%, 38.16%, and 44.28%, respectively. Moreover, a reduction of 45.83% for machine downtime and 29.51% for rejected outputs/waste appears to be crucial. To achieve 100% efficiency for the entire production system, a comprehensive approach involving cutbacks in all input factors and undesirable outputs, along with an increment of 31.28% in good outputs, is essential.

### The proposed framework of NDEA-based PMS

Managers find the NDEA-based PMS to be effective in analysing the performance fluctuations of production factors and outputs in each production process. This sheds light on the impact of such fluctuations on the overall performance of the production line. Both the production manager and supervisors concurred that the NDEA model comprehensively addressed the essential measures related to medical device manufacturing operations. The model facilitated identifying inefficient processes and pinpointed the production factors or outputs that required enhancement. Decisions associated with process enhancement typically fall in the purview of the manufacturing head department or production line managers and supervisors.

**Figure 5.**
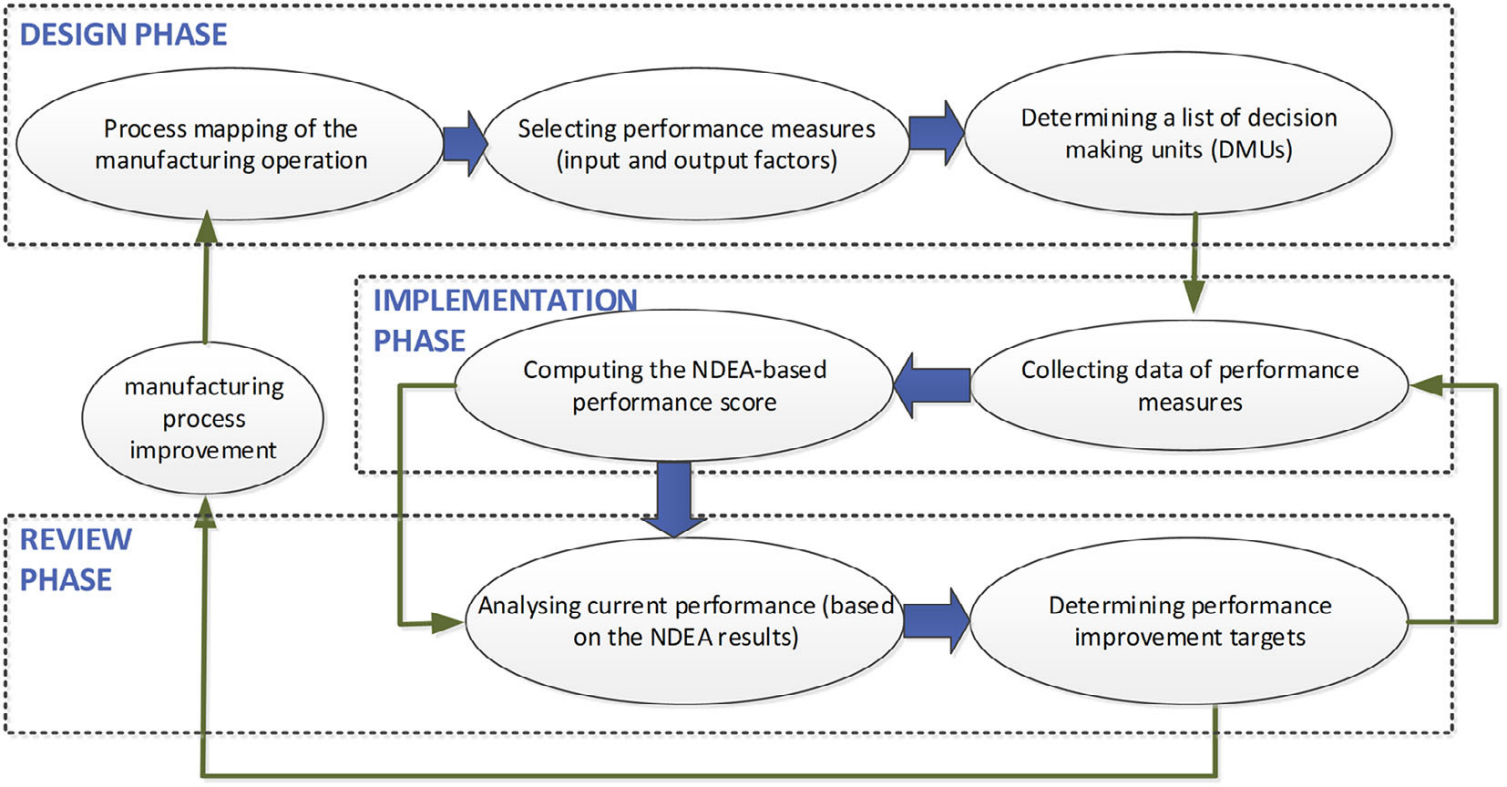
Proposed NDEA-based PMS framework. The diagram outlines three main phases: Design (process mapping and model selection), Implementation (data collection and scoring), and Review (adjustments and benchmarking).

A post-study meeting with the company’s executive board emphasized the need for a generic framework to extend DEA applications in manufacturing. A generalized framework is essential for managers applying similar techniques across production lines or manufacturing companies. Aligning with PMS principles, the proposed framework consists of three main phases: design, implementation, and review (see Figure 5).

In the design phase, the initiation involves process mapping, outlining the systematic flow of the manufacturing process from raw materials to finished products ( Lindsay et al., 2003). A process map visually portrays the systematic flow of sub-processes from start to finish ( Wilson, 2004). According to ( Sinclair & Zairi, 1995a, 1995b) performance measures are classified into inputs, processes, and outputs. The NDEA-based PMS defines input and output factors for each subprocess involved in the production line, enabling efficiency evaluation, benchmarking, and process improvement.

Regular assessments of the production system’s performance—daily, weekly, or monthly—are crucial during the implementation phase. Data collection precedes the calculation of efficiency scores using NDEA software. The model outcomes provide performance scores for each DMU, identifying top performers. For underperforming DMUs, the NDEA model pinpoints sub-processes causing inefficiencies.

In the review phase, two scenarios arise: for major modifications affecting the entire manufacturing process, the cycle resets to the start of the framework. For minor process changes, revisiting the last two stages—implementation and review—is sufficient. Minor alterations involve reviewing improvement targets and benchmarking against peer groups to align with the best performers.

## Results and discussion

### 1. Efficiency scores and peer groups

The study assessed the IV-set production line’s performance using five NDEA models, focusing on a three-stage model for practical insights. Due to its single-case design, formal statistical validation was not conducted. Efficiency scores showed that 50% of production months (DMUs) were inefficient, providing guidance for process improvement. Inefficient DMUs were benchmarked against efficient peers, such as comparing production months 02_23, 07_23, and 11_23 to target reductions in material waste, machine downtime, and operator-related issues. This highlights NDEA’s ability to identify specific inefficiencies, unlike classical DEA, which treats the production line as a “black box” ( Lotfi et al., 2023; Tone & Tsutsui, 2009).

### 2. Stage-specific insights

The stage-level analysis uncovered substantial variation in efficiency across the production line, revealing the following patterns:

• PVC Granulation (WS1): The least efficient stage, with the largest performance variability. Inefficiencies were driven primarily by raw material quality, machine downtime, and operator variability.
• Moulding (WS2–3): Moderate efficiency with intermediate variability; inefficiencies were related to subassembly quality and coordination issues.
• Assembly (WS4–6): Highest average efficiency and minimal variability, indicating a stabilizing effect on overall production.

These insights provide practical diagnostic guidance for managers, emphasizing that improving WS1 would significantly enhance overall production efficiency. This finding aligns with prior manufacturing studies, which often show early-stage processes dominate total inefficiency ( Castelli et al., 2010; Ma et al., 2025).

### 3. Trade-offs between stage granularity and discrimination power

A comprehensive comparison of all five NDEA formulations highlights an inherent methodological tension in network modelling. As models incorporate greater stage granularity to more accurately reflect the complexity of real production systems, they also risk introducing dimensionality challenges, redundancy across variables, and sensitivity to data variability. These factors can ultimately weaken a model’s ability to discriminate between efficient and inefficient DMUs. Conversely, more consolidated network structures may enhance discrimination but at the cost of losing important process-level insights. The results of this study show a clear trade-off between retaining detailed system representation and maintaining analytical robustness:

• **Six-stage NDEA:** Provided detailed process-level insights but classified a large share of DMUs as efficient, limiting its ability to differentiate performance.
• **Three-stage NDEA:** Achieved the most balanced performance, preserving essential granularity while still enabling meaningful differentiation across DMUs.
• **One- and two-stage NDEA:** Oversimplified the production process, missing important intermediate transformations.

These outcomes demonstrate that network granularity increases the variable count while the DMU sample size remains fixed, thereby inflating degrees of freedom and compressing the efficiency frontier, thereby reducing discrimination ( Akbarian, 2021; Asanimoghadam et al., 2022). Correlated stages or redundant intermediate outputs can further dilute discrimination, causing DMUs to appear efficient and making it harder to distinguish true performance differences ( Amini et al., 2021). Model orientation, distance form, and inclusion of undesirable outputs also affect frontier shape and efficiency interpretation ( Tone & Tsutsui, 2009; Zuniga-Gonzalez et al., 2025).

### 4. Process improvement targets

The NDEA model generated slacks for each input and output, translating inefficiency into actionable targets: Material consumption: Reduce by 45.08%, Man-hours: Reduce by 38.16%, Machine-hours: Reduce by 44.28%, Machine downtime: Reduce by 45.83%, Rejected outputs/waste: Reduce by 29.51%, Desirable outputs: Increase by 31.28%. These stage-specific targets allow managers to prioritize improvements, such as optimizing machine schedules, enhancing PVC granulation quality, and improving operator training in WS1. This highlights NDEA’s value in decision support and continuous monitoring, which classical DEA alone cannot provide.

### 5. Practical implications

The findings of this study offer several actionable insights that can guide decision-makers, practitioners, and industry stakeholders in improving performance and refining management practices. These implications are outlined as follows:

• **Strategic stage selection**: Strategic stage selection is crucial, with managers advised to balance stage design to capture key interdependencies without compromising discrimination power and group processes with minimal variability.
• **Targeted improvement**: By adopting the NDEA-based PMS, managers can focus on the most inefficient stages and their sources of inefficiency. For example, in the IV-set production line, optimizing machine schedules and improving raw material quality in WS1 could significantly enhance overall efficiency.
• **Cross-industry applicability**: The proposed NDEA-based PMS framework provide a potential applicability to other industries with complex, multi-stage production systems, such as other industries with complex, multi-stage production systems, like automotive or electronics, to identify bottlenecks and improve throughput.
• **Data-driven decision-making**: The NDEA-based PMS allows data-driven decision-making. Hence **,** regularly updating efficiency scores and monitoring peer groups can help managers identify emerging inefficiencies and adapt processes accordingly.

### 6. Theoretical contributions

By integrating methodological insights with practical modelling considerations, this study deepens and refines the theoretical understanding of performance measurement in multi-stage production systems. The key theoretical contributions are as follows:

• **Development of Practical Framework:** The study contributes to the performance measurement literature by presenting of a practical framework for implementing NDEA-based PMS in manufacturing settings. This framework provides structured guidance for managers on stage selection and process consolidation to enhance discrimination power without sacrificing granularity. It emphasizes the importance of benchmarking and continuous monitoring to drive process improvements and efficiency gains.
• **Discrimination Power of NDEA Models:** The study also contribute to the DEA/NDEA literature, by revealing that while increasing the number of stages provides greater granularity and insights into specific processes, it reduces the discrimination power of the model. The six-stage model, for instance, classified a disproportionately high number of Decision-Making Units (DMUs) as efficient, limiting its utility for pinpointing inefficiencies. In contrast, the three-stage model offered a balance between granularity and discrimination power, making it the most practical choice for performance evaluation. We reconcile this apparent conflict by highlighting several interlocking explanations consistent with the literature:
  ○ *Dimensionality and sample size effects*: Increasing network granularity raises the number of variables while keeping the DMU sample size fixed, potentially inflating degrees of freedom and compressing the frontier, thus reducing discriminatory power. This issue is well-documented in DEA studies as model complexity increases relative to sample size ( Akbarian, 2021; Asanimoghadam et al., 2022).
  ○ *Correlation and redundancy among stages*: When stages are highly correlated or intermediate products offer little unique information, adding more stages can dilute discrimination despite increasing parameterization, as decomposition is effective only when sub-processes provide distinct insights ( Cantor & Poh, 2020).
  ○ *Orientation, distance form, and scale choices*: Using a non-oriented, non-radial CRS NSBM and considering undesirable outputs alters the frontier’s shape compared to radial models, affecting inefficiency dimensions and potentially leading to divergent discrimination outcomes ( Tone & Tsutsui, 2009; Zuniga-Gonzalez et al., 2025).
  ○ *Data quality and noise amplification*: More complex network models can exacerbate measurement errors, reducing the ability to differentiate performance levels. Although managing undesirable data can help, it does not completely resolve this issue ( Lotfi et al., 2023; J. Wu et al., 2017).

## Conclusion

This paper contributes to the manufacturing PMS research domain in several ways. First, it initiates the integration of NDEA into PMS for a manufacturing process, thereby developing a practical framework termed “NDEA-based PMS”. Second, the case study investigating the application of NDEA in a pharmaceutical production line shed light on the intricacies of the shop floor by modelling performance indicators for a multi-stage production line and highlighting the relevance of NDEA for manufacturing performance measurement and process improvement. The practical framework proposed from the insights of the case study, has answered RQ1, expanding the application of NDEA, highlighting its ability to decompose production stages and providing insightful information for strategic decision-making.

Beyond this methodological contribution, the study yields two substantive empirical findings that merit careful comparison with existing literature. First, the application of a non-oriented, non-radial NSBM (CRS) that explicitly accounts for desirable and undesirable outputs produced robust stage-level efficiency estimates and practical diagnostic information consistent with prior work showing the value of network and slack-based approaches for multi-stage and environmentally-sensitive settings ( Lotfi et al., 2023; Ma et al., 2025; Tone & Tsutsui, 2009).

Second, and more unexpectedly, our comparison across five NDEA formulations indicates that increasing the number of explicitly modelled stages reduced the discrimination power of efficiency scores in this case study (RQ2). This result contradicts claims in some prior contributions that NDEA generally increases discrimination relative to classical DEA ( Castelli et al., 2010). The inconsistency in discrimination outcomes across NDEA formulations can be attributed to several factors. As network granularity increases, more variables are introduced while DMUs remain constant, inflating degrees of freedom and compressing the efficiency frontier, which reduces discriminatory power. Highly correlated stages add parameters without improving differentiation. Model choices, such as non-oriented CRS NSBM structures and including undesirable outputs, also alter the efficiency frontier and influence inefficiency identification. Furthermore, increased model complexity can heighten data noise and measurement errors, affecting efficiency accuracy.

Despite its contributions, this study is not without limitations. The proposed NDEA-based PMS framework, developed and applied within a single pharmaceutical production line, requires further validation before it can be confidently generalized to other manufacturing contexts, especially given the substantial variation in industrial environments and process configurations. The empirical analysis is based on a relatively small dataset—12 months of observations representing 12 DMUs—which is adequate for illustrative purposes but insufficient for formal statistical validation or hypothesis testing. Moreover, data availability and quality constraints, combined with assumptions of homogeneity within production units and the use of static, linear modelling structures, may oversimplify the dynamic and heterogeneous nature of real operational systems. Confidentiality restrictions further limited the level of detail that could be disclosed regarding actual inputs and outputs, reducing transparency and full replicability. Addressing these limitations in future research would strengthen the robustness of the findings and support broader applicability of the proposed framework.

Future research should focus on external validation of the NDEA-based PMS framework across diverse manufacturing contexts and industries to enhance generalizability. Researchers may implement various methods to investigate the weights and relationships between performance measures. Exploring the dynamics of the manufacturing system over time is a promising avenue. Dynamic NDEA models may capture changes in efficiency, process interactions, and improvement targets to offer a more comprehensive view of the evolving manufacturing landscape.

In conclusion, this research bridges a significant gap in the literature by integrating NDEA into a practical PMS framework, addressing the unique challenges of multi-stage manufacturing systems. The model’s capacity to grant detailed visibility and flexibility qualifies it as a transformative tool for researchers and practitioners. Future research is advised to persist in aligning operational practice with strategic intent and continue to serve the NDEA-based PMS framework as a portal to long-term competitiveness and efficiency in the complicated and globalized manufacturing landscape.

## Ethics statement

This study did not involve human participants, human tissue, or personally identifiable information. The analysis was conducted using anonymized internal production data obtained from a private manufacturing firm under a confidentiality agreement. As such, the study falls outside the scope of research involving human subjects as defined by the Declaration of Helsinki, and formal approval by an Institutional Review Board (IRB) or ethics committee was not required.

Nevertheless, the research protocol and data use were reviewed and approved by the Research Ethics Committee of the author’s institution to ensure compliance with institutional ethical standards and data governance requirements. No permit or reference number was issued, as the study did not involve human subjects or data requiring formal ethical clearance.

## Data Availability

The dataset used in this study comprises confidential internal production data obtained from a private manufacturing firm. Due to the sensitive nature of the data and the confidentiality agreement in place, the dataset cannot be made publicly available. Sharing the data would risk disclosing proprietary information and commercially sensitive operational details. This research involved no human subjects, and therefore did not require formal Institutional Review Board (IRB) approval. However, the data access and use were reviewed and approved by the research team’s affiliated institution to ensure compliance with ethical and contractual obligations. Researchers interested in accessing the dataset for verification or replication purposes may submit a formal request to the corresponding author. Access may be granted under specific conditions, including the signing of a non-disclosure agreement (NDA) and written approval from the data provider. All such requests will be evaluated on a case-by-case basis in accordance with the data provider’s confidentiality policies.
