# Supplementary material for: Enhancing Efficiency in Multi-Stage Pharmaceutical Manufacturing: A Process-Based Network DEA Approach
Source: F1000Res. 2025 Dec 4;14:710. Originally published 2025 Jul 18. [Version 2] doi: 10.12688/f1000research.166387.2 (PMC13080329; doi:10.12688/f1000research.166387.2)
Supplement: Peer review history

# Reviewer report: Reviewer response for version 2

**DOI:** 10.5256/f1000research.192568.r472794

**Published:** 2026-04-21

**Authors:** Hayder Mohammed Issa

**Affiliations:** 1 University of Garmian, Kalar, Iraq

The manuscript demonstrates significant academic and practical value by employing a non-oriented, CRS-NSBM Network Data Envelopment Analysis to disaggregate the traditional "black-box" manufacturing model, thereby providing actionable, stage-specific insights into the production of intravenous sets. This approach is particularly commendable for its integration of undesirable outputs, such as machine downtime and rejected products, which allows for a more realistic appraisal of operational inefficiencies, specifically identifying PVC granulation as the most volatile and inefficient stage.

 While the research effectively navigates the inherent trade-off between model granularity and discrimination power—rightly favoring the three-stage model for its balanced analytical robustness—the work could be further strengthened by addressing the constraints of the 12-month sample size, perhaps through the future consideration of higher-frequency weekly data to enhance statistical discrimination. Furthermore, acknowledging how the substantial potential improvements identified, such as the proposed 45% reduction in material consumption, would interface with the stringent validated state and regulatory requirements typical of pharmaceutical environments would offer a more nuanced perspective for practitioners. Notwithstanding these minor, optional considerations for future refinement, the paper’s methodological rigor and its successful bridging of performance measurement theory with industrial application make it a reasonable contribution to the field, and I recommend its acceptance as it stands.

Is the case presented with sufficient detail to be useful for teaching or other practitioners?

Yes

Is the work clearly and accurately presented and does it cite the current literature?

Yes

If applicable, is the statistical analysis and its interpretation appropriate?

Yes

Are all the source data underlying the results available to ensure full reproducibility?

Partly

Are the conclusions drawn adequately supported by the results?

Yes

Is the background of the case’s history and progression described in sufficient detail?

Yes

Reviewer Expertise:

Process Engineering, Pharmaceutical Processing, Process Design & Development. Process Simulation & Modelling

I confirm that I have read this submission and believe that I have an appropriate level of expertise to confirm that it is of an acceptable scientific standard.

# Reviewer report: Reviewer response for version 2

**DOI:** 10.5256/f1000research.192568.r469679

**Published:** 2026-04-14

**Authors:** Dr. Santhosh Illendula

**Affiliations:** 1 Vijaya College of Pharmacy, Hayathnagar, Telangana, India

The author has described case study report satisfactory

 Author has well explained the study with Interaction of inputs, intermediate factors, and outputs in five NDEA models, Interaction among input, output, and intermediate factors in two-stage manufacturing operations. NDEA optimizes multi-stage pharmaceutical manufacturing by "opening the black box" of production to analyze, measure, and improve individual stages (e.g., synthesis, formulation, packaging) rather than just the final output.

 It identifies specific inefficiency sources, handles intermediate product transfers, enables better resource allocation, and supports data-driven decisions. These case study report has been satisfactory and accepted further process, Thank you

Is the case presented with sufficient detail to be useful for teaching or other practitioners?

Yes

Is the work clearly and accurately presented and does it cite the current literature?

Yes

If applicable, is the statistical analysis and its interpretation appropriate?

Yes

Are all the source data underlying the results available to ensure full reproducibility?

Yes

Are the conclusions drawn adequately supported by the results?

Yes

Is the background of the case’s history and progression described in sufficient detail?

Yes

Reviewer Expertise:

Department of Pharmaceutical Analysis ( Pharmacy)

I confirm that I have read this submission and believe that I have an appropriate level of expertise to confirm that it is of an acceptable scientific standard.

# Reviewer report: Reviewer response for version 2

**DOI:** 10.5256/f1000research.192568.r438579

**Published:** 2025-12-26

**Authors:** Kassu Jilcha

**Affiliations:** 1 College of Technology and Built Environment, Addis Ababa, Addis Ababa, Ethiopia

Dears,

 The paper now has shown major improvement and if the case is needed, please try to restructure the paper as Introduction, Literature review (alone standing title), Methodology, Result and Discussion and Conclusion.

 Regards

Is the case presented with sufficient detail to be useful for teaching or other practitioners?

Partly

Is the work clearly and accurately presented and does it cite the current literature?

Partly

If applicable, is the statistical analysis and its interpretation appropriate?

Partly

Are all the source data underlying the results available to ensure full reproducibility?

Partly

Are the conclusions drawn adequately supported by the results?

Partly

Is the background of the case’s history and progression described in sufficient detail?

Partly

Reviewer Expertise:

Innovation and industrial engineering areas

I confirm that I have read this submission and believe that I have an appropriate level of expertise to confirm that it is of an acceptable scientific standard.

# Reviewer report: Reviewer response for version 1

**DOI:** 10.5256/f1000research.183368.r405838

**Published:** 2025-09-24

**Authors:** Rashed Ahmed

**Affiliations:** 1 North South University, Dhaka, Dhaka Division, Bangladesh

**Summary of the Article**

 The manuscript presents a case study applying **Network Data Envelopment Analysis (NDEA)** to a pharmaceutical manufacturing line (intravenous (IV) sets) to build a process-based Performance Measurement System (PMS). The authors review DEA and NDEA theory and then describe the IV-set production process across six workstations. They collect 12 months of production data and compare five modeling scenarios (treating the process as 1 to 6 stages). Efficiency scores (DMU per month) are computed for each model; descriptive statistics show how many months are classified as “efficient.” From this analysis, the authors select a three-stage model (combining certain processes) as the preferred balance of discrimination power and detail. Using that model, they identify that the **PVC granulation stage (Stage 1)** is the least efficient (highest waste and downtime), whereas the final assembly stage is the most efficient. The NDEA yields slacks (shortfalls) indicating that large reductions in inputs (materials, labor, machine-hours) and undesirable outputs (rejects, downtime) would be needed to reach full efficiency. Based on these insights, the authors propose a practical NDEA-based PMS framework: it guides managers on how to select stages, benchmark against peer production months, monitor efficiency scores over time, and iteratively refine processes. The paper concludes that this framework “integrates NDEA into a practical PMS” and highlights its potential for guiding multi-stage manufacturing improvements and strategic decisions.

 The manuscript provides basic context about the pharmaceutical company (product lines, market share, regulatory environment) and maps the IV-set production stages. However, it lacks depth on the operational history or baseline performance issues motivating the study (e.g. why inefficiencies arose, any past improvements). Additional details (e.g. initial performance metrics, company’s maturity or prior interventions) would strengthen the case context.The overall narrative is understandable, and the paper cites many relevant sources (including recent 2022–2025 studies). However, the literature review omits several modern developments (e.g. recent advances in NDEA, bootstrap DEA, Industry 4.0 performance measurement) and some citations are outdated. The writing and notation have inconsistencies (e.g. merged words, unclear figures, table references), which occasionally impede clarity. Improved copy-editing, figure/table clarity, and inclusion of newer references would greatly enhance accuracy and readability.The study uses a non-parametric efficiency analysis (NDEA) without traditional statistical hypothesis tests or confidence intervals. The interpretation relies on descriptive statistics of efficiency scores. While NDEA itself is appropriate for the research question, the manuscript does not perform any statistical validation (e.g. bootstrap confidence intervals or tests) to support claims about differences between models or stages. Adding formal statistical validation methods would strengthen the analysis.The data are proprietary and require an NDA for access. The paper states that data are confidential, so independent researchers cannot verify the results. For reproducibility, the authors should provide (or detail) sufficient data summaries, synthetic datasets, or clear data processing steps. At minimum, comprehensive summary statistics or sample data should be supplied.The core findings (e.g. the 3-stage model’s balance between detail and discrimination, PVC granulation as the least efficient stage) align with the presented results. However, some claims exceed what the data show. For example, the assertion that this framework “bridges a significant gap” or that conclusions “contradict classical DEA” are overstated given the single-case analysis. The conclusions generalize broadly (to other industries or strategic decision-making) without validation. Toning down these claims and clearly linking conclusions to specific results would make them more defensible.The production process is well-mapped and described, and key efficiency scores are reported. Yet the case lacks certain practical details (e.g. actual input/output quantities, exact improvement actions taken, implementation challenges) that would benefit practitioners. For teaching purposes, more numerical examples or step-by-step illustrations of the NDEA application (beyond abstract descriptions) would improve usefulness.

**Comments to the Authors**

 Thank you for addressing an important industrial problem by applying NDEA to pharmaceutical manufacturing. The case study is interesting, and the idea of integrating NDEA into a performance system is promising. However, the manuscript in its present form has multiple issues that must be addressed to make the study scientifically sound and useful. Below are the main concerns and suggestions:

1. **Sample Size and DEA Validity:** You analyze only 12 DMUs (months) with many inputs and outputs (including intermediate flows). Standard DEA practice requires much larger samples (often 2–3× the total number of variables) to obtain reliable efficiency scores. With so few observations, nearly half the months are “efficient” by default. This undermines your conclusions about discrimination power. **Suggestions:**
  - If more data are available (e.g. additional years or multiple production lines), include them to boost DMU count.
  - Alternatively, simplify the model by reducing variables (e.g. combine some similar inputs or outputs) so that the 12 observations are sufficient.
  - Clearly acknowledge this limitation in the text as a **pilot study** with limited generalizability. Avoid making broad claims based on this small sample.
2. **Statistical Validation:** The current comparisons between models (1-stage vs 3-stage, etc.) are based solely on descriptive statistics (means, standard deviations). There is no measure of uncertainty. **Suggestions:**
  - Use DEA bootstrap methods to compute confidence intervals for efficiency scores and test differences between models.
  - Report p-values or other statistical tests (e.g. Friedman test) to support claims that one model discriminates better than another.
  - Add sensitivity analysis: show how results change with small data perturbations or alternate variable selections.
3. **Model Selection Justification:** The choice of the three-stage model is based on a qualitative “parsimony” argument, but this is not rigorous. **Suggestions:**
  - Define explicit criteria for selecting the preferred model (for instance, minimize the number of efficient DMUs while still capturing all key processes).
  - Consider formal model selection metrics (analogous to AIC, BIC) or cross-validation if possible.
  - Explain why three stages reflect the real production better than two or four stages. Consult with production engineers to validate the stage grouping (the reader should see why combining certain stages makes sense).
4. **Data Transparency:** The data are proprietary, but for scientific reporting, we need some level of transparency. **Suggestions:**
  - Provide summary statistics (means, ranges) for each input and output variable.
  - If possible, anonymize and share data (even if synthetic with a similar structure) as supplementary material.
  - At a minimum, explicitly list all inputs/outputs by stage (not just symbolic names in Table 3). Briefly describe how each was measured.
5. **Case and Context Details:** The narrative lacks detail on the real-world context and how the study was motivated or used. **Suggestions:**
  - Quantify the performance gaps that prompted this study (e.g. known waste rates, downtime percentages prior to analysis).
  - Explain why the 12-month period was chosen (seasonality issues? data availability?).
  - Describe how managers or operators participated: was the NDEA analysis actually implemented or tested in operations?
  - If any follow-up actions were taken (process changes, investments) as a result of this analysis, briefly mention them.
6. **Literature Review and References:** Some relevant recent work appears to be missing. **Suggestions:**
  - Include citations for modern NDEA applications and improvements (e.g. dynamic/network DEA, bootstrap DEA methods, DEA in healthcare or manufacturing post-2020).
  - Mention any case studies of NDEA in pharmaceuticals or medical devices if available.
  - Update or clarify references that seem only tangentially related (e.g. if a study is on general DEA in banking, explain relevance).
7. **Presentation and Clarity:** There are multiple formatting and clarity issues. **Suggestions:**
  - Carefully proofread: fix typos and spacing (e.g. “therewas,” “developingNDEA-based,” etc.).
  - Standardize notation: ensure all variables (x, y, z) and subscripts are consistent and clearly defined (some appear cut or incomplete in tables/figures).
  - Improve figures/tables: For example, Figure 3 (NDEA model diagrams) needs clear labels and captions; Table 5 should have a complete caption and readable formatting.
  - In the text, clearly refer to all figures and tables at first mention and summarize their key points in words.
8. **Results Interpretation:** The discussion should more directly connect to the literature and avoid overclaiming. **Suggestions:**
  - When stating that “this contradicts classical DEA,” clarify whether you ran a classical DEA or are inferring from others’ claims. If you did classical DEA (1-stage model), present those results side-by-side.
  - Frame findings as preliminary insights (“in this case, we found…”) rather than definitive rules.
  - Compare your results with any similar studies (if available). Discuss whether, for instance, identifying the first stage as inefficient agrees with known industry benchmarks.
9. **Conclusions and Claims:** Several claims go beyond the data. **Suggestions:**
  - Tone down statements about generalizability (e.g. “applicable to automotive/electronics”). Instead, suggest these as potential extensions with future validation.
  - Reiterate study limitations (sample size, single site, confidentiality constraints) explicitly in the conclusion.
  - Clearly link each major conclusion back to specific results shown (for example, “Based on Table 4, models with more stages had higher average efficiency scores and more efficient DMUs, indicating lower discrimination”).

By addressing the points above, the manuscript will be much stronger. In particular, focus on bolstering the methodological rigor (sample size issue, statistical validation) and ensuring claims are commensurate with what a single-case analysis can support. With these revisions, the paper will better serve practitioners interested in using NDEA for process improvement.

Is the case presented with sufficient detail to be useful for teaching or other practitioners?

Partly

Is the work clearly and accurately presented and does it cite the current literature?

Partly

If applicable, is the statistical analysis and its interpretation appropriate?

Partly

Are all the source data underlying the results available to ensure full reproducibility?

No

Are the conclusions drawn adequately supported by the results?

Partly

Is the background of the case’s history and progression described in sufficient detail?

Partly

Reviewer Expertise:

I am an operations researcher with applied expertise in Data Envelopment Analysis (including network and slacks-based models), statistical validation of frontier methods (bootstrap inference), and the design and evaluation of Performance Measurement Systems in manufacturing environments. My applied experience includes process mapping, efficiency improvement, and operational decision support in regulated manufacturing (pharmaceuticals/medical devices).

I confirm that I have read this submission and believe that I have an appropriate level of expertise to confirm that it is of an acceptable scientific standard, however I have significant reservations, as outlined above.

## References

[1] : A network Data Envelopment Analysis to estimate nations’ efficiency in the fight against SARS-CoV-2. *Expert Systems with Applications* .2022;210: 10.1016/j.eswa.2022.118362 10.1016/j.eswa.2022.118362 PMC935574735958804

[2] : Approach in inputs & outputs selection of Data Envelopment Analysis (DEA) efficiency measurement in hospitals: A systematic review. *PLOS ONE* .2024;19(8) : 10.1371/journal.pone.0293694 10.1371/journal.pone.0293694 PMC1132414439141630

[3] : Adapting Efficiency Analysis in Health Systems: A Scoping Review of Data Envelopment Analysis Applications During the COVID-19 Pandemic. *Journal of Market Access & Health Policy* .2024;12(4) : 10.3390/jmahp12040024 306-316 10.3390/jmahp12040024 PMC1150328939464180

[4] : The use of Data Envelopment Analysis (DEA) in healthcare with a focus on hospitals. *Health Care Management Science* .2019;22(2) : 10.1007/s10729-018-9436-8 245-286 10.1007/s10729-018-9436-8 29478088

[5] : Efficiency of health resource utilisation in primary-level maternal and child health hospitals in Shanxi Province, China: a bootstrapping data envelopment analysis and truncated regression approach. *BMC Health Services Research* .2020;20(1) : 10.1186/s12913-020-5032-y 10.1186/s12913-020-5032-y 32143651 PMC7059375

[6] : Levels, trends, and determinants of effectiveness on the hierarchical medical system in China: Data envelopment analysis and bootstrapping truncated regression analysis. *Frontiers in Public Health* .2022;10: 10.3389/fpubh.2022.921303 10.3389/fpubh.2022.921303 PMC953044836203685

[7] : Primary Care Efficiency Measurement Using Data Envelopment Analysis: A Systematic Review. *Journal of Medical Systems* .2015;39(1) : 10.1007/s10916-014-0156-4 10.1007/s10916-014-0156-4 25486892

[8] : Performance Measurement System and Quality Management in Data-Driven Industry 4.0: A Review. *Sensors* .2021;22(1) : 10.3390/s22010224 10.3390/s22010224 35009767 PMC8749653

# Response

**Published:** 2025-12-04

**Authors:** Mahmood Shubbak

**Affiliations:** Sultan Qaboos University, Muscat, Muscat Governorate, Oman

**RESPONSE TO REVIEWER #2: Rashed Ahmed**

 Comments to the Authors Thank you for addressing an important industrial problem by applying NDEA to pharmaceutical manufacturing. The case study is interesting, and the idea of integrating NDEA into a performance system is promising. However, the manuscript in its present form has multiple issues that must be addressed to make the study scientifically sound and useful. Below are the main concerns and suggestions:

**1- Sample Size and DEA Validity:** You analyze only 12 DMUs (months) with many inputs and outputs (including intermediate flows). Standard DEA practice requires much larger samples (often 2–3× the total number of variables) to obtain reliable efficiency scores. With so few observations, nearly half the months are “efficient” by default. This undermines your conclusions about discrimination power. **Suggestions:**

- If more data are available (e.g. additional years or multiple production lines), include them to boost DMU count.
- Alternatively, simplify the model by reducing variables (e.g. combine some similar inputs or outputs) so that the 12 observations are sufficient.
- Clearly acknowledge this limitation in the text as a **pilot study** with limited generalizability. Avoid making broad claims based on this small sample.

**Response:** We thank you for highlighting the critical issue of sample size and its implications for the validity and discrimination power of DEA/NDEA models. We acknowledge that the small sample size limits statistical generalizability.

 The case study is intended as an **illustrative example** of our proposed NDEA-based PMS framework. We clarified this in the Introduction, Methodology, and Conclusion. To mitigate potential overclaiming, we also emphasized the descriptive and exploratory nature of the results rather than statistical inference.

 This article works under category of case study paper, thus the case is intentionally designed as an **illustrative example, **or **demonstrative application** of our proposed NDEA-based PMS framework. To follow up your note, we have revised the final part of the introduction as follows:

 “To answer these questions, this article presents a case study focused on the production line of a pharmaceutical company's intravenous (IV) sets, exemplifying the intricacies of multi-stage manufacturing, featuring a combination of manual and automated processes. The case study is intentionally illustrative; the goal is to demonstrate the feasibility and practical utility of a NDEA-based PMS rather than to generate statistically generalizable results. Using a case study of intravenous (IV) set production, the research illustrates how NDEA can be operationalized to:

- Provide a practical framework for routine efficiency monitoring and process improvement.
- Identify stage-specific inefficiencies as the focus of process improvement.
- Evaluate trade-offs between model granularity and discrimination power;

By embedding model selection criteria, incorporating stage validation with production engineers, and accounting for undesirable outputs, the study provides a robust methodology for implementing NDEA in operational settings. In doing so, it bridges the theoretical development of network DEA with practical performance management needs in complex manufacturing environments.”

**2- Statistical Validation:** The current comparisons between models (1-stage vs 3-stage, etc.) are based solely on descriptive statistics (means, standard deviations). There is no measure of uncertainty. **Suggestions:**

- Use DEA bootstrap methods to compute confidence intervals for efficiency scores and test differences between models.
- Report p-values or other statistical tests (e.g. Friedman test) to support claims that one model discriminates better than another.
- Add sensitivity analysis: show how results change with small data perturbations or alternate variable selections.

**Response:** We thank you for raising the critical point regarding the statistical validation. Due to the limited DMU sample, formal bootstrap methods and statistical tests were not applied. Our study focuses on developing the practical NDEA-based PMS framework. To follow up your suggestion, we have clarified this limitation in the Methodology and Discussion.

 To address the reviewer’s concern, we have revised the manuscript to:

- In introduction: Explicitly state that the aim of the study is methodological framework development rather than statistical inference, in introduction, as addressed in point 1.
- In methodology: Explicit statement in the methodology that that statistical validation is not feasible with the available sample.
- In conclusion: Acknowledge that bootstrap DEA and statistical testing represent valuable extensions, but are not feasible given the illustrative nature and limited data availability of the case setting. Suggest these techniques as directions for future research, particularly when larger datasets are available to support inferential analysis and sensitivity testing.

**3- Model Selection Justification:** The choice of the three-stage model is based on a qualitative “parsimony” argument, but this is not rigorous. **Suggestions:**

- Define explicit criteria for selecting the preferred model (for instance, minimize the number of efficient DMUs while still capturing all key processes).
- Consider formal model selection metrics (analogous to AIC, BIC) or cross-validation if possible.
- Explain why three stages reflect the real production better than two or four stages. Consult with production engineers to validate the stage grouping (the reader should see why combining certain stages makes sense).

**Response: **We thank you for highlighting the need for a more rigorous justification of the chosen three-stage model. We agree that a qualitative parsimony argument alone is insufficient and have revised the manuscript accordingly.

 In the revised version of our **methodology**: We have adopted an explicit criterion for model selection: the preferred model minimizes the number of efficient DMUs while capturing all key production processes, aligning with your suggestion. Additionally, we have strengthened the justification for the three-stage structure by incorporating consultation with production engineers from the participating pharmaceutical manufacturer. Their expert input confirmed that the three-stage grouping most accurately reflects the functional and operational flow of the IV-set production line. In particular, they indicated that:

- A two-stage model would oversimplify the process and obscure necessary intermediate transformations.
- A four-stage model would fragment stages that are tightly integrated in practice, offering limited additional managerial insight.

**4- Data Transparency:** The data are proprietary, but for scientific reporting, we need some level of transparency. **Suggestions:**

- Provide summary statistics (means, ranges) for each input and output variable.
- If possible, anonymize and share data (even if synthetic with a similar structure) as supplementary material.
- At a minimum, explicitly list all inputs/outputs by stage (not just symbolic names in Table 3). Briefly describe how each was measured.

**Response: **We thank you for emphasizing the importance of data transparency in scientific reporting. While we fully agree with the need for sufficient clarity to ensure reproducibility, we regret that the **raw operational data cannot be shared** due to strict confidentiality agreements with the participating pharmaceutical manufacturer. These data contain commercially sensitive production information that cannot be disclosed publicly, even in anonymized or synthetic form, under the terms of our partnership.

 To address the reviewer’s concern while respecting these constraints, we have provided the manuscript to strengthen transparency in the following ways:

- **Detailed variable listing:** We have expanded the description of the variables by explicitly listing all inputs, outputs, and intermediate flows by stage.
- **Measurement explanations:** For each variable, we now provide a brief explanation of how it is measured in the production process to ensure clarity and interpretability for readers.
- **Clarification of confidentiality limitations:** We explicitly state in the text that raw or shareable datasets cannot be provided due to proprietary restrictions.

These revisions ensure that the methodology and variable structure are fully transparent, allowing readers to understand and interpret the model even without access to the protected dataset.

**5- Case and Context Details:** The narrative lacks detail on the real-world context and how the study was motivated or used. **Suggestions:**

-
  - Quantify the performance gaps that prompted this study (e.g. known waste rates, downtime percentages prior to analysis).
  - Explain why the 12-month period was chosen (seasonality issues? data availability?).
  - Describe how managers or operators participated: was the NDEA analysis actually implemented or tested in operations?
  - If any follow-up actions were taken (process changes, investments) as a result of this analysis, briefly mention them.

**Response:** We thank the reviewer for highlighting the need to provide more contextual details and practical motivation for the case study. We have revised the manuscript to clarify the real-world context, quantify performance gaps, and describe managerial participation. We have added these details in the **Case Study section (Section 5)** to provide readers with a clearer understanding of the real-world context, performance gaps, and managerial involvement.

**6. Literature Review and References:** Some relevant recent work appears to be missing. **Suggestions:**

- Include citations for modern NDEA applications and improvements (e.g. dynamic/network DEA, bootstrap DEA methods, DEA in healthcare or manufacturing post-2020).
- Mention any case studies of NDEA in pharmaceuticals or medical devices if available.
- Update or clarify references that seem only tangentially related (e.g. if a study is on general DEA in banking, explain relevance).

**Response: ** Thank you for your valuable feedback. To follow up, we revised our manuscript (in the **introduction and literature review sections**) by incorporating ****citations of recent (post-2020) applications of network/decomposed DEA, including dynamic/network DEA, bootstrap methods, and hybrid approaches in manufacturing and supply chain contexts. This situates our work at the current frontier of efficiency measurement research and clarifies our choice of non-radial, non-oriented NSBM CRS over dynamic or bootstrap methods.

 Meanwhile, we reviewed literature on NDEA applications in pharmaceuticals and medical devices, highlighting this emerging area. When no prior studies were found, we noted the gap and positioned our case study as an early contribution. We also clarified non-manufacturing DEA references by adding sentences that explain their relevance, focusing on methodological insights and discrimination power. We added more relevant references, as in the earlier note, to enhance the coherence of the literature review.

**7. Presentation and Clarity:** There are multiple formatting and clarity issues. **Suggestions:**

- Carefully proofread: fix typos and spacing (e.g. “therewas,” “developingNDEA-based,” etc.).
- Standardize notation: ensure all variables (x, y, z) and subscripts are consistent and clearly defined (some appear cut or incomplete in tables/figures).
- Improve figures/tables: For example, Figure 3 (NDEA model diagrams) needs clear labels and captions; Table 5 should have a complete caption and readable formatting.
- In the text, clearly refer to all figures and tables at first mention and summarize their key points in words.

**Response:** We appreciate the reviewer’s careful attention to presentation and clarity. In response, we have undertaken a thorough review of the manuscript and implemented the improvements through the entire manuscript.

**8. Results Interpretation:** The discussion should more directly connect to the literature and avoid overclaiming. **Suggestions:**

- When stating that “this contradicts classical DEA,” clarify whether you ran a classical DEA or are inferring from others’ claims. If you did classical DEA (1-stage model), present those results side-by-side.
- Frame findings as preliminary insights (“in this case, we found…”) rather than definitive rules.
- Compare your results with any similar studies (if available). Discuss whether, for instance, identifying the first stage as inefficient agrees with known industry benchmarks.

**Response:** We thank the reviewer for highlighting the need for more careful interpretation of the results and closer linkage to the literature. In response, we have revised the entire structure of Results and Discussion.

**9. Conclusions and Claims:** Several claims go beyond the data. **Suggestions:**

- Tone down statements about generalizability (e.g. “applicable to automotive/electronics”). Instead, suggest these as potential extensions with future validation.
- Reiterate study limitations (sample size, single site, confidentiality constraints) explicitly in the conclusion.
- Clearly link each major conclusion back to specific results shown (for example, “Based on Table 4, models with more stages had higher average efficiency scores and more efficient DMUs, indicating lower discrimination”).

**Response:** We appreciate the reviewer for highlighting the importance of moderating claims and clarifying the limitations of our study. We have revised the Conclusion section accordingly. Specifically, we have: Softened statements regarding the generalizability of the results in both the results and discussion sections. Clearly reiterated the study's limitations, including sample size, being conducted at a single site, and confidentiality constraints, in the limitations section of the conclusion.

# Response

**Published:** 2026-03-10

**Authors:** Mahmood Shubbak

**Affiliations:** Sultan Qaboos University, Muscat, Muscat Governorate, Oman

We have addressed your comments in the revised version of our manuscript. Please check it.

# Reviewer report: Reviewer response for version 1

**DOI:** 10.5256/f1000research.183368.r405835

**Published:** 2025-09-10

**Authors:** Kassu Jilcha

**Affiliations:** 1 College of Technology and Built Environment, Addis Ababa, Addis Ababa, Ethiopia

Abstract Enhancement: The abstract should incorporate a clear statement regarding the ordinality or significance of this paper within the broader field. It is essential to articulate how this research contributes to existing knowledge, highlighting its unique value and relevance.

 Introduction Citations: The introduction section requires the inclusion of citations from recent studies published in 2025. This will help position the current research within the most up-to-date context, demonstrating engagement with the latest developments in the field.

 Citation Style Improvement: The citation style currently utilized, such as (Färe & Primont, 1984), should be revised. It is recommended to remove the brackets around the authors’ names and to place the publication year within brackets. This change will enhance readability and align the citations with standard practices in academic writing.

 Literature Review Depth: The literature review appears to be somewhat superficial. It is important to enrich this section with references to recent studies to provide a more comprehensive overview of the current state of research. Incorporating more contemporary sources will strengthen the foundation of this paper.

 Results and Discussion Expansion: The results and discussion section lacks depth and requires a more detailed comparison of the findings with prior studies. A thorough analysis that juxtaposes current results with existing literature will provide a clearer understanding of the implications and significance of the research outcomes.

Is the case presented with sufficient detail to be useful for teaching or other practitioners?

Partly

Is the work clearly and accurately presented and does it cite the current literature?

Partly

If applicable, is the statistical analysis and its interpretation appropriate?

Partly

Are all the source data underlying the results available to ensure full reproducibility?

Partly

Are the conclusions drawn adequately supported by the results?

Partly

Is the background of the case’s history and progression described in sufficient detail?

Partly

Reviewer Expertise:

Innovation and industrial engineering areas

I confirm that I have read this submission and believe that I have an appropriate level of expertise to confirm that it is of an acceptable scientific standard, however I have significant reservations, as outlined above.

# Response

**Published:** 2025-12-04

**Authors:** Mahmood Shubbak

**Affiliations:** Sultan Qaboos University, Muscat, Muscat Governorate, Oman

**AUTHORS' RESPONSE TO REVIEWER #1: Kassu Jilcha**

**Comment #1:**

**Abstract Enhancement: The abstract should incorporate a clear statement regarding the ordinality or significance of this paper within the broader field. It is essential to articulate how this research contributes to existing knowledge, highlighting its unique value and relevance.**

**Response;** We appreciate your feedback. The significance of the paper has been incorporated into the conclusion section of the abstract as follows:

 “This study holds significance within the broader field of performance measurement and efficiency analysis by bridging theoretical modelling and practical implementation. It advances existing knowledge through the integration of NDEA into a process-based PMS, offering a novel analytical framework for multi-stage manufacturing systems. By examining the trade-off between model complexity and discrimination power, this research contributes new methodological insights and extends the applicability of NDEA in real-world industrial settings. The framework offers managers actionable guidance for optimizing multi-stage manufacturing operations and contributes novel insights into the methodological behaviour of NDEA. Ultimately, this work strengthens the linkage between performance measurement theory and industrial practice, positioning NDEA as a valuable tool for continuous improvement in manufacturing systems.”

**Comment #2:**

**Introduction Citations: The introduction section requires the inclusion of citations from recent studies published in 2025. This will help position the current research within the most up-to-date context, demonstrating engagement with the latest developments in the field.**

**Response:** thank you for your feedback. We have added some updated citations, for example at the end of the first paragraph” Robust and flexible performance measurement tools are not just administrative necessities, they are strategic enablers that help organizations navigate financial burdens, enhance competitiveness, and foster the innovation and market dynamics needed for future success (Calik, 2024; Xing et al., 2025; Xu & Zhu, 2024).“ and also at the beginning of the third paragraph.

**Comment #3:**

**Citation Style Improvement: The citation style currently utilized, such as (Färe & Primont, 1984),should be revised. It is recommended to remove the brackets around the authors’ names and to place the publication year within brackets. This change will enhance readability and align the citations with standard practices in academic writing.**

**Response;** Thank you for your correction. We have updated the citation style, specifically by removing the brackets around the author’s names at the beginning of sentences and placing the publication year within brackets. This adjustment is reflected in the literature review: “The Basic Concept of NDEA: Färe & Primont (1984) initiated the exploration of the 'black-box' system of classical DEA”

**Comment #4:**

**Literature Review Depth: The literature review appears to be somewhat superficial. It is important to enrich this section with references to recent studies to provide a more comprehensive overview of the current state of research. Incorporating more contemporary sources will strengthen the foundation of this paper.**

**Response:** Thank you for this valuable comment. We agree that strengthening the depth and currency of the literature review will improve the overall foundation of the paper. In the revised manuscript, we have expanded the literature review to incorporate a broader range of recent and relevant studies, for example ”Recent research has advanced the treatment of intermediate and undesirable outputs, refining NDEA models to better capture complex network structures, dual-role factors, and process interdependencies. For example, Lotfi et al. (2023) applied an NDEA model to assess both desirable and undesirable outputs in the wheat supply chain, demonstrating enhanced accuracy in identifying stage-specific inefficiencies. Ma et al. (2025) proposed a network slack-based measure incorporating dual-role factors and undesirable outputs to evaluate supply chain performance, highlighting the practical relevance of NDEA in complex production networks. (Yang et al., 2024) further demonstrate the flexibility of NDEA by incorporating shared resources, negative data, and undesirable outputs in a multi-stage airline efficiency context, highlighting its capacity to model interdependencies realistically.  This development allows decision-makers to simultaneously optimize performance while reducing waste or other negative byproducts, thereby providing a more nuanced and actionable understanding of operational efficiency. Essentially, NDEA’s ability to model undesirable outputs transforms efficiency assessment into a more realistic and strategically valuable instrument for complex production systems.

 These revisions provide a more comprehensive overview of the current research landscape and better contextualize the contribution of our work. We appreciate your guidance, which has helped us enhance the rigour and relevance of this section.

# Reviewer report: Reviewer response for version 1

**DOI:** 10.5256/f1000research.183368.r405839

**Published:** 2025-09-03

**Authors:** pravin Ullagaddi

**Affiliations:** 1 University of the Cumberlands, Williamsburg, Kentucky, USA

This manuscript presents a case study applying Network Data Envelopment Analysis (NDEA) to evaluate performance in pharmaceutical manufacturing, specifically focusing on intravenous (IV) set production. The authors analyze a single pharmaceutical company's production line across six interconnected workstations over 12 monthly periods, comparing five different NDEA model configurations (1-stage through 6-stage). They conclude that a 3-stage model provides the optimal balance between analytical detail and discrimination power, and propose a generic framework for implementing NDEA-based Performance Measurement Systems (PMS) in manufacturing environments.

 The study identifies the PVC granulation stage as the primary source of inefficiency and recommends substantial reductions in inputs and undesirable outputs. The authors claim their framework contributes to manufacturing PMS literature and provides actionable guidance for multi-stage production systems.

 Critical Assessment

 Scientific Soundness Issues (Must Be Addressed)

 1. Fundamental Sample Size Violation

 The most critical flaw is the use of only 12 Decision Making Units (DMUs) with models containing up to 15+ variables. This violates the basic DEA requirement that sample size should be at least twice the number of inputs plus outputs. This violation renders all efficiency scores and model comparisons unreliable.

 Required remediation:

- Expand the dataset to include at least 36 monthly observations, or
- Reduce model complexity to maximum 6 total variables, or
- Include multiple production lines/facilities to increase DMU count, or
- Acknowledge this as a pilot study with limited generalizability

2. Absence of Statistical Validation

 The paper presents efficiency scores and model comparisons without confidence intervals, significance tests, or uncertainty measures. Claims about discrimination power differences rely solely on descriptive statistics.

 Required remediation:

- Implement bootstrap methodology to generate confidence intervals for all efficiency scores
- Conduct statistical hypothesis tests for model comparison claims
- Add sensitivity analysis for variable selection and model specification
- Report p-values for claimed differences between models

3. Inadequate Model Selection Justification

 The selection of the 3-stage model as "optimal" lacks rigorous criteria. The authors cite parsimony and discrimination power but provide no statistical framework for this critical decision.

 Required remediation:

- Develop and apply formal model selection criteria (AIC, cross-validation, etc.)
- Test multiple variable combinations within each stage configuration
- Provide theoretical justification for why 3 stages best represent the production system
- Validate the stage structure with process engineers and production experts

Literature and Presentation Issues

 4. Outdated and Incomplete Literature Review

 The literature review misses significant recent developments in NDEA methodology and manufacturing performance measurement, relying heavily on foundational papers from the 1980s-2000s.

 Recommended improvements:

- Conduct systematic review of NDEA applications in manufacturing (2015-2024)
- Include recent advances in bootstrap DEA, dynamic NDEA, and two-stage models
- Cover pharmaceutical-specific performance measurement frameworks
- Address Industry 4.0 and digital manufacturing measurement approaches

5. Poor Presentation Quality

 Mathematical notation is inconsistent, figures are unclear, and writing quality impedes comprehension.

 Recommended improvements:

- Redesign Figure 3 with clear visual hierarchy and consistent labeling
- Standardize mathematical notation throughout
- Provide comprehensive copyediting for grammar and clarity
- Add detailed captions explaining all figure elements

Data and Reproducibility Concerns

 6. Complete Data Unavailability

 All source data is confidential and inaccessible, preventing independent verification or replication.

 Recommended approaches:

- Create synthetic datasets that preserve analytical relationships
- Provide detailed data generation procedures for replication
- Partner with other manufacturers to create multi-site validation
- At minimum, provide detailed descriptive statistics for all variables

7. Insufficient Case Context

 The background lacks detail necessary for understanding the organizational setting, problem severity, and implementation context.

 Required additions:

- Quantify baseline performance problems that motivated the study
- Describe the company's operational maturity and previous improvement initiatives
- Explain the 12-month study period selection and any external influences
- Document stakeholder engagement and validation processes
- Report actual implementation outcomes and organizational responses

Methodological Limitations

 8. Weak Theoretical Foundation

 The paper treats NDEA application as a primarily technical exercise without grounding in operations management theory or pharmaceutical manufacturing strategy.

 Recommended strengthening:

- Connect the analysis to manufacturing strategy frameworks
- Justify variable selection based on operations theory
- Link findings to broader pharmaceutical industry performance challenges
- Integrate regulatory compliance considerations into the analysis

9. Overstated Conclusions

 Claims about generalizability, framework novelty, and practical impact far exceed what the limited analysis supports.

 Required modifications:

- Restrict conclusions to the specific case studied
- Acknowledge the pilot nature of the study
- Remove claims about broad industry applicability without validation
- Present the framework as preliminary rather than proven

Constructive Recommendations

 For Immediate Revision:

1. Expand the empirical foundation by including additional time periods, production lines, or partner organizations to achieve adequate sample size
2. Implement statistical rigor through bootstrap confidence intervals, hypothesis testing, and sensitivity analysis
3. Strengthen case documentation with detailed organizational context, problem quantification, and implementation outcomes
4. Moderate conclusions to reflect the study's limitations and preliminary nature

For Long-term Research Development:

1. Multi-site validation across different pharmaceutical manufacturers to test framework generalizability
2. Longitudinal analysis tracking performance improvements over extended periods following NDEA implementation
3. Comparative methodology study evaluating NDEA against alternative performance measurement approaches in manufacturing contexts
4. Integration research examining how NDEA-based PMS interfaces with existing ERP/MES systems and organizational processes

Verdict

 This manuscript addresses a relevant practical problem but suffers from fundamental methodological flaws that compromise its scientific validity. The sample size violation alone disqualifies the statistical analysis, while the absence of validation, weak theoretical foundation, and overstated conclusions further limit its contribution.

 Recommendation: Major revision is required with particular attention to the sample size issue, statistical validation, and conclusion moderation. The authors should consider repositioning this as a preliminary pilot study rather than a definitive framework development, with clear acknowledgment of limitations and need for further validation.

 The pharmaceutical manufacturing community would benefit from rigorous research in this area, but this work requires substantial strengthening to meet academic indexing standards and provide reliable guidance to practitioners.

Is the case presented with sufficient detail to be useful for teaching or other practitioners?

Partly

Is the work clearly and accurately presented and does it cite the current literature?

No

If applicable, is the statistical analysis and its interpretation appropriate?

No

Are all the source data underlying the results available to ensure full reproducibility?

No

Are the conclusions drawn adequately supported by the results?

No

Is the background of the case’s history and progression described in sufficient detail?

Partly

Reviewer Expertise:

Manufacturing Systems, Statistical analyses, Process Optimization, Pharmaceutical Manufacturing

I confirm that I have read this submission and believe that I have an appropriate level of expertise to state that I do not consider it to be of an acceptable scientific standard, for reasons outlined above.

# Response

**Published:** 2026-03-12

**Authors:** Mahmood Shubbak

**Affiliations:** Sultan Qaboos University, Muscat, Muscat Governorate, Oman

We have revised our manuscript. Kindly check the new version. Also kindly note that its category is Case Study.
